# Novel and sustainable microfabricated Cu ion selective sensor doped with ionophore and supported with docking study for determination of vonoprazan fumarate in tablet dosage form

**DOI:** 10.1186/s13065-025-01714-9

**Published:** 2026-01-30

**Authors:** Helal Zaher, Emad Ramzy, Amr M. Mahmoud, Mona S. Elshahed, Radwan EL-Haggar

**Affiliations:** 1 Pharmaceutical Chemistry Department, Faculty of Pharmacy, Capital University (formerly Helwan University), Ein Helwan, Cairo, 11795 Egypt; 2 Analytical Chemistry Department, Faculty of Pharmacy, Capital University (formerly Helwan University), Ein Helwan, Cairo, 11795 Egypt; 3 Analytical Chemistry Department, Faculty of Pharmacy, Cairo University, Kasr El-Aini Street, Cairo, 11562 Egypt

**Keywords:** Vonoprazan fumarate, Ion-selective electrode, Microfabrication, Graphene nanocomposite, Greenness assessment, Molecular docking

## Abstract

In the field of drug analysis, there is a growing emphasis on developing techniques that are environmentally friendly, cost-effective, and efficient. To align with the principles of green analytical chemistry and to support the advancement of portable and handheld devices, an innovative microfabricated ion selective electrode (ISE) has been developed for the detection of Vonoprazan fumarate (VON). The development of this electrode involved a two-step optimization process. Initially, a range of ionophores were screened to determine the one with the highest selectivity for VON. Through molecular docking studies, gamma-cyclodextrin (γ-cyclodextrin) was identified as demonstrating maximal activity towards VON. The second optimization step involved incorporating a graphene nanocomposite as an ion to electron transducer layer between the γ-cyclodextrin polymeric membrane and the microfabricated copper (Cu) solid contact ISE. This nanocomposite layer contributed to enhanced stability, reduced potential drift, and rapid response times (approximately 30 s), likely due to its hydrophobic properties that prevent water layer formation at the interface between the Cu electrode and the polymeric membrane. The VON sensor was characterized according to IUPAC guidelines, revealing a linear dynamic range of 2.00 × 10⁻^5^ to 1.00 × 10⁻^2^ M (equivalent to 9.23 to 4615.00 µg/mL) and a limit of detection (LOD) of 1.00 × 10⁻^5^ M. This sensor was successfully utilized for the selective determination of VON in bulk powder and pharmaceutical formulations. Statistical analysis showed no significant difference when comparing the results with those obtained using the reported method. The environmental impact of the method was assessed using Complex-GAPI and BAGI tools.

**Graphical Abstract:**

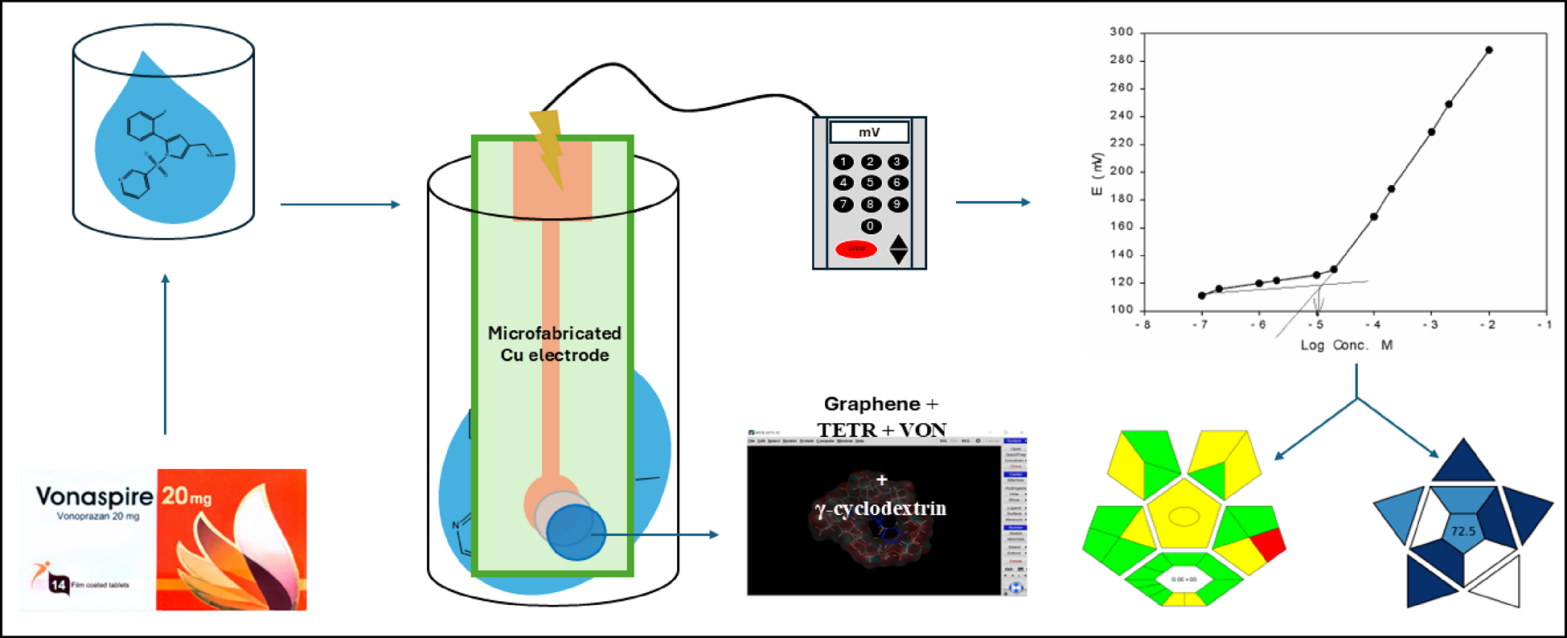

**Supplementary Information:**

The online version contains supplementary material available at 10.1186/s13065-025-01714-9.

## Introduction

Vonoprazan fumarate (VON), chemically known as 1-Methyl-4-(2-(2-((methyl sulfonyl)methyl) phenyl) thiazol-4-yl) methyl) pyridin-1-ium fumarate, is a potassium-competitive acid blocker that reversibly inhibits the Hydrogen/potassium adenosine triphosphatase (H⁺/K⁺-ATPase) enzyme in gastric parietal cells, leading to potent and sustained suppression of gastric acid secretion. It is approved for the treatment of acid-related diseases, including gastroesophageal reflux disease, peptic ulcers, and Helicobacter pylori infections [1]. Unlike traditional proton pump inhibitors, VON does not require acid activation, providing a rapid onset of action and prolonged acid suppression, even in patients with cytochrome P450 2C19 polymorphisms [2]. Additionally, VON has demonstrated a favorable safety profile, with fewer drug interactions and a lower risk of adverse effects compared to proton pump inhibitors [2]. These pharmacological advantages position VON as a superior therapeutic option for managing acid-related disorders. Several analytical techniques have been developed for the determination of VON, each with its unique advantages and limitations. Such as chromatographic techniques [3–9], Spectrofluorometric methods [10–13], and electrochemical methods [14, 15]. Although the previously mentioned methods have reasonable sensitivity for the studied drug, they have some limitations. The chromatographic methods including LC–MS/MS and HPLC methods are complex, time-consuming, and require expensive instrumentation, making them less accessible for routine analysis in resource limited countries [3–5]. Additionally, environmental concerns have been raised regarding the use of organic solvents and energy-intensive procedures in some methods [6, 11]. These limitations highlight the need for more sustainable, efficient, and cost-effective analytical techniques. Although several electrochemical methods have been reported using carbon paste as the working electrode, the manual preparation of homemade carbon paste electrodes often leads to inconsistent surface characteristics. This variability in electrode composition and texture can compromise performance and adversely affect the reproducibility of the obtained results [15].

Potentiometric ion-selective electrodes (ISEs) inherently align with the core principles of green chemistry and sustainable analytical practices, providing a compelling alternative to conventional methods, this is evident through their operational characteristics, which substantially reduce the environmental footprint often associated with techniques like HPLC [3, 4, 16]. ISEs support sustainability by requiring minimal or no hazardous organic solvents, operating under ambient conditions (low energy consumption), and often allowing for direct measurement in complex matrices with minimal sample preparation [17]. Furthermore, the development of microfabricated all-solid-state ISEs, such as the one described herein, promotes miniaturization and portability, aligning with the "waste prevention" and "design for energy efficiency" principles of green chemistry. This commitment to sustainable methodology is a crucial factor in contemporary analytical research, often evaluated using advanced metrics like the Complex-GAPI and BAGI [18, 19]. The use of ISEs for pharmaceutical analysis has been successfully demonstrated, highlighting their applicability as fast, economic, simple, and sensitive analytical tools [17].

Traditional ISEs consist of a polyvinyl chloride (PVC) membrane separating two solutions, an inner reference solution with a fixed analyte concentration and a sample solution with variable analyte concentrations. The membrane potential is measured using Ag/AgCl reference electrodes. However, classical ISEs have limitations, including their large size, large sample volume requirements, and the need for specialized construction skills [18]. To address these drawbacks, solid-state ISEs have emerged as a promising alternative as the ion selective membrane (ISM) is directly interfaced with a solid transducer, eliminating the need for an inner filling solution [20, 21]. Recently, there has been growing interest in mass-producing cost-effective, portable, and reliable all-solid-state ISEs for pharmaceutical and environmental analyses [22]. Such electrodes have been successfully applied for the rapid and economical detection of pharmaceuticals, food additives [23–25], and biologically effective compounds [25].

Cu is a cost-effective alternative to gold or platinum electrodes and is compatible with microfabrication and printed circuit board (PCB) processes [26]. Cu wire electrodes paired with HPLC enable potentiometric detection through analyte interaction with the Cu surface, generating a potential via oxidation/reduction [27]. Cu electrodes are promising for microfabrication While they generally face limitations such as oxidation during current passage, this issue is avoided in potentiometric measurements [28]. However, Cu based solid contact ISEs suffer from unstable potentials and high drift due to poor ion to electron transduction [29]. An aqueous layer at the solid contact membrane interface may act as an electrolyte reservoir, affecting stability and LOD [29–31]. To enhance performance, hydrophobic interlayers such as carbon nanotubes [32], conducting polymers [30], or graphene [33] can improve electron transfer. Graphene is a highly conductive, thermally stable material with a large surface area, making it ideal for sensors and nanocomposites [34]. It serves as an efficient ion to electron transducer, offering fast charge transfer, chemical stability, mechanical strength, and hydrophobicity [32, 35–39]. The modification of potentiometric sensors using lipophilic ionophore allows for better discrimination in the electrode's selectivity towards the target analyte. This is beneficial for interference studies and enhancing the electrode's sensitivity. Prior work has employed techniques such as molecular docking to predict the optimal ionophores for the analyte by analyzing their potential interactions, orientations, and fitting [40, 41].

Microfabricated Cu electrodes offer significant advantages over homemade carbon paste electrodes, particularly in terms of reproducibility, stability, and sensitivity in potentiometric drug determination. Their precise manufacturing ensures uniform dimensions, surface properties, and electrochemical behavior, leading to highly reproducible measurements essential for accurate drug analysis [21, 42]. In contrast, the manual preparation of carbon paste electrodes introduces variability, resulting in inconsistent performance. Stability is another crucial factor, as Cu electrodes exhibit superior chemical and durability, maintaining their properties over time, whereas carbon paste electrodes tend to degrade due to the soft and malleable nature of the paste, negatively impacting long-term reproducibility [41]. Furthermore, microfabricated electrodes are produced using standardized processes, ensuring consistency and minimizing human error, while homemade carbon paste electrodes require manual preparation, which can introduce inconsistencies. Cu electrodes also offer greater flexibility in surface modification, allowing for enhanced selectivity toward specific drugs, whereas carbon paste electrodes have limited modification options. Although microfabricated Cu electrodes may have a higher initial cost, their durability and reproducibility reduce the need for frequent replacements, making them cost-effective in the long term [21, 42–44]. Overall, the superior reproducibility, stability, and sensitivity of microfabricated Cu electrodes make them a more reliable choice for potentiometric drug determination over the classical carbon paste electrode [15, 43, 44]. Accordingly, this study focuses on developing validated and reliable microfabricated Cu ISEs that integrate lipophilic ionophores for the quantification of VON in tablet dosage form. Molecular docking was employed to select the appropriate ionophore, in addition to the microfabricated Cu electrodes were modified with graphene as an efficient ion to electron transducer, offering fast charge transfer, chemical stability, mechanical strength, and hydrophobicity to prevent the aqueous layer formation and consequently to enhance the LOD.

## Experimental

### Materials and reagents

VON pure drug was kindly supplied by Jiangxi Synergy Pharmaceutical Co., Ltd (China), and its purity was certified to be not less than 98.00% according to supplier specifications. Vonaspire® tablets (26.72 mg vonoprazan fumarate eq. to 20.0 mg vonoprazan), manufactured by Inspire Pharma, were purchased from the local market in Egypt. PVC of high molecular weight, dibutyl phthalate (DBP), ortho-nitrophenyl octyl ether (o-NPOE), dioctyl phthalate (DOP), potassium tetrakis(4-chlorophenyl) borate (TETR), γ-cyclodextrin, tetrahydrofuran (THF), sodium hydroxide (NaOH) and hydrophobic graphene nanoplatelets powder were purchased from Sigma-Aldrich (St. Louis, USA). Phosphotungstic acid (PTA) was purchased from Fluka (Switzerland). Pre-sensitized printed circuit boards were obtained from M.G. Chemicals (Ontario, Canada). Sodium tetraphenylborate (TPB) was obtained from Oxford Lab Fine Chem (India). Monobasic sodium phosphate was from Riedel-de Haën AG (Hannover, Germany). All reagents are of analytical grade, and all experiments were conducted using double-distilled water.

### Instrumentation

All measurements were conducted at 25 °C using Adwa digital pH/mV and Temperature Meter (model AD1030, Romania) equipped with Ag/AgCl double-junction reference electrode (Steinhum, Germany). To investigate the effect of pH on the electrode's performance, a pH glass electrode (Adwa, Romania) was utilized. The measurements were carried out with the assistance of a magnetic stirrer (Bandelin Sonorox, Budapest, Hungary). Scanning Electron Microscopy (Quanta FEG250). Energy-Dispersive X-ray Spectroscopy (OCTAN PRO).

### Microfabrication of Cu electrodes

The Cu electrodes were microfabricated following a published method [45]. In short, a photomask with precise electrode dimensions was created using CAD software and printed onto a transparent sheet with a high-resolution laser printer. This pattern was then transferred onto photoresist-coated printed circuit boards (PCBs) by UV light exposure (360 nm, 30 s). The CAD-designed photomask was placed over the PCB, shielding selected areas from UV light while leaving others exposed. Next, the photoresist region was developed using a 0.25 M NaOH solution to dissolve the exposed regions. The Cu etching step was carried out with a 1.10 M NH₄S₂O₈ solution at 40 °C. After etching, acetone was used to strip away the remaining photoresist, revealing the Cu electrode pattern. The electrodes were then rinsed with acetone and glacial acetic acid to eliminate Cu oxide residues, followed by a final water wash to ensure a clean surface.

### Preparation of ISM

To identify the optimal combination of ion pair (IP) and plasticizer, several ISMs were prepared without ionophore by dissolving the membrane components in 3.0 mL of THF. γ-cyclodextrin was chosen as the ionophore according to the molecular docking study outcomes. Different amounts of γ-cyclodextrin were then incorporated into the optimized membrane formulation (as summarized in Table 1). Each resulting solution was drop-cast onto solid contact electrode and left overnight to allow complete evaporation of the solvent.

**Table 1:** Percentage composition of materials in the sensing layer of various sensors

Sensor	% Composition			
	IP	Plasticizer	γ-cyclodextrin	PVC
PTA				
Sensor 1	3.20%	64.50% (DOP)	–	32.30%
Sensor 2	3.20%	64.50% (DBP)	–	32.30%
Sensor 3	3.20%	64.50% (o-NPOE)	–	32.30%
TETR				
Sensor 4	3.20%	64.50% (DOP)	–	32.30%
Sensor 5	3.20%	64.50% (DBP)	–	32.30%
Sensor 6	3.20%	64.50% (o-NPOE)	–	32.30%
Sensor 7	1.60%	65.50% (o-NPOE)	–	32.80%
Sensor 8	4.80%	63.50% (o-NPOE)	–	31.70%
Sensor 9	3.13%	62.50% (o-NPOE)	3.13%	31.25%
Sensor 10	3.17%	63.50% (o-NPOE)	1.59%	31.74%
Sensor 11	3.08%	61.54% (o-NPOE)	4.62%	30.77%
Sensor 12 (doped with graphene nanoplate)	3.13%	62.50% (o-NPOE)	3.13%	31.25%

### Preparation of graphene nanocomposite

**Scheme 1:**
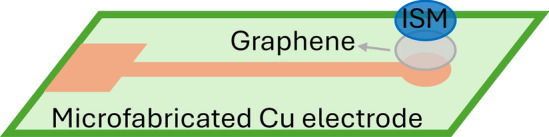
Schematic representation of microfabricated Cu electrode

The graphene/PVC nanocomposite (10%) was prepared [46] as follows: Graphene sheets were dispersed in 1.0 mL of xylene via sonication. Separately, PVC was dissolved in 3.0 mL of THF with the addition of 200.0 µL o-NPOE as a plasticizer. The two solutions were then mixed and stirred for 10 min. The resulting graphene/PVC composite was deposited via drop-casting (15.0 μL) onto a microfabricated Cu electrode surface. The solvent was allowed to evaporate for 24 h to minimize mixing between the transducer and membrane layers, which could compromise long-term stability. Next, 15.0 μL of the ISM (Sensor 9) was drop-casted onto the electrode, forming the Cu graphene-based ISE (Sensor 12, as illustrated in Scheme 1). To evaluate the effect of the nanocomposite graphene on potential stability, a control experiment was conducted using a Cu-graphene-free solid contact electrode (Sensor 9), which was tested under identical conditions (water layer and stability tests). Before measurements, the solid-contact ISEs were left overnight at room temperature to allow THF evaporation, followed by soaking in a 1.00 × 10⁻^4^ M VON solution overnight.

### Calibration graph

The microfabricated electrodes were preconditioned by immersing them in a 0.01 M VON solution 2 h prior to measurements. Subsequently, the electrodes, along with a double-junction Ag/AgCl reference electrode, were submerged in working solutions of VON with concentrations ranging from 1.00 × 10⁻⁶ to 1.00 × 10⁻^2^ M. These working solutions were prepared in a 0.02 M phosphate buffer adjusted to a pH of 5.00 ± 0.05. Between measurements, the microfabricated electrodes were rinsed with double-distilled water. All measurements were conducted under constant stirring. The electromotive forces (emfs) were recorded as a function of VON concentration, and calibration curve was plotted by drawing the measured potential against the logarithm of VON concentrations.

### Preparation of the stock solution for tablets dosage form

Seven Vonaspire® tablets were individually weighed, and the average weight of the tablets was calculated. The tablets were then transferred to a clean, dry mortar and ground into a fine powder. An accurately weighed portion of the powder, equivalent to 24.27 mg of VON, was transferred to a 50 mL volumetric flask containing 30.0 mL of phosphate buffer (pH 5.00 ± 0.05). The mixture was sonicated for 20 min in an ultrasonic bath at 25 °C. After sonication, the solution was diluted to the mark with the same buffer and filtered through a 0.45 μm disposable syringe filter. The resulting solution was labeled to have a concentration of 1.00 × 10^−3^ M VON. Subsequently, a diluted solution was prepared with a claimed concentration of 5.00 × 10^−5^ M, this final concentration was determined using the selected sensor (Sensor 12) and the results were compared with the reported HPLC method [7].

### Determination of selectivity coefficients

The selectivity coefficients of the electrode were determined using the separate-solution method [47]. In this approach, the cell potentials of both the primary analyte solution (*E*_i_) and an equimolar (1 × 10^−2^ M) interfering ion solution (*E*_j_) were measured. The selectivity coefficient (log *K*^pot^_i,j_) was then computed based on the Nicolsky-Eisenman equation:

$$ \begin{aligned} {\mathrm{Log}}K_{{{\mathrm{i}},{\mathrm{j}}}}^{{{\mathrm{pot}}.}} =\, & \left( {E_{j} - E_{i} } \right)z_{i} F/2.303RT \\ & + \left( {1 - \left( {z_{i} /z_{j} } \right)} \right){\text{ Log}}a_{i} \\ \end{aligned} $$

where: E_i_ and E_j_ are the potentials of primary and interfering ions, respectively, R is the gas constant, T is the temperature, z_i_ and z_j_ are the charges of the primary and interferent ions, respectively, and F is the faraday constant.

### Molecular docking

Using the MOE 2015.10 software, the potential interactions between various ionophores including calix[4]arene, calix[6]arene, calix[8]arene, and the cyclodextrins (α-, β-, and γ), were explored in relation to VON drug. In this study, each ionophore was treated as the receptor, while VON compound was considered the ligand. Both ionophores and the ligand were prepared in a virtual environment by protonating them at pH 5.00, followed by energy minimization to stabilize their structures. The next step involved examining possible binding conformations between each receptor and the ligand using the triangle matcher algorithm. The resulting docking poses were ranked based on their docking scores [40, 41]. Additionally, the software was utilized to visualize the interactions within the selected complex and to generate a graphical representation of the receptor's binding pocket with the bound ligand.

## Results and discussion

### Screening and optimization of ISEs

#### Effect of IP and plasticizer type

The screening phase serves as a preliminary step preceding the optimization process, particularly when multiple variables are under investigation. Its primary purpose is to model the system's response and identify the experimental parameters that yield optimal analytical performance. In this study, screening experiments were conducted by systematically varying individual factors specifically the type of plasticizer, the IP, and their ratios within the ISM while maintaining all other conditions constant. This approach was employed to assess the influence of each variable on the ISMs sensitivity and the resulting slope of the response. During this stage, two different pairing agents, PTA and TETR were evaluated. Both achieved a Nernstian slope; however, TETR exhibited a higher slope along with a more stable potential (Sensors 3, 5, and 6). Additionally, membrane cocktails incorporating o-NPOE, DBP, or DOP were investigated. The lowest slope was observed with DOP, while o-NPOE provided the best overall response (Sensors 1–6). When mixed with TETR (Sensor 6), the o-NPOE notably improved the slope of the calibration curve, increasing it from 47.00 to 52.18 mV/decade.

#### Optimization of IP amount

Subsequently different amounts of TETR (Sensor 6–8) were examined to determine its optimal amount. The results revealed that 10.0 mg of TETR is the optimal amount that produced the highest Nernstian response (Sensor 6).

#### Ionophore selection via molecular docking

**Fig. 1:**
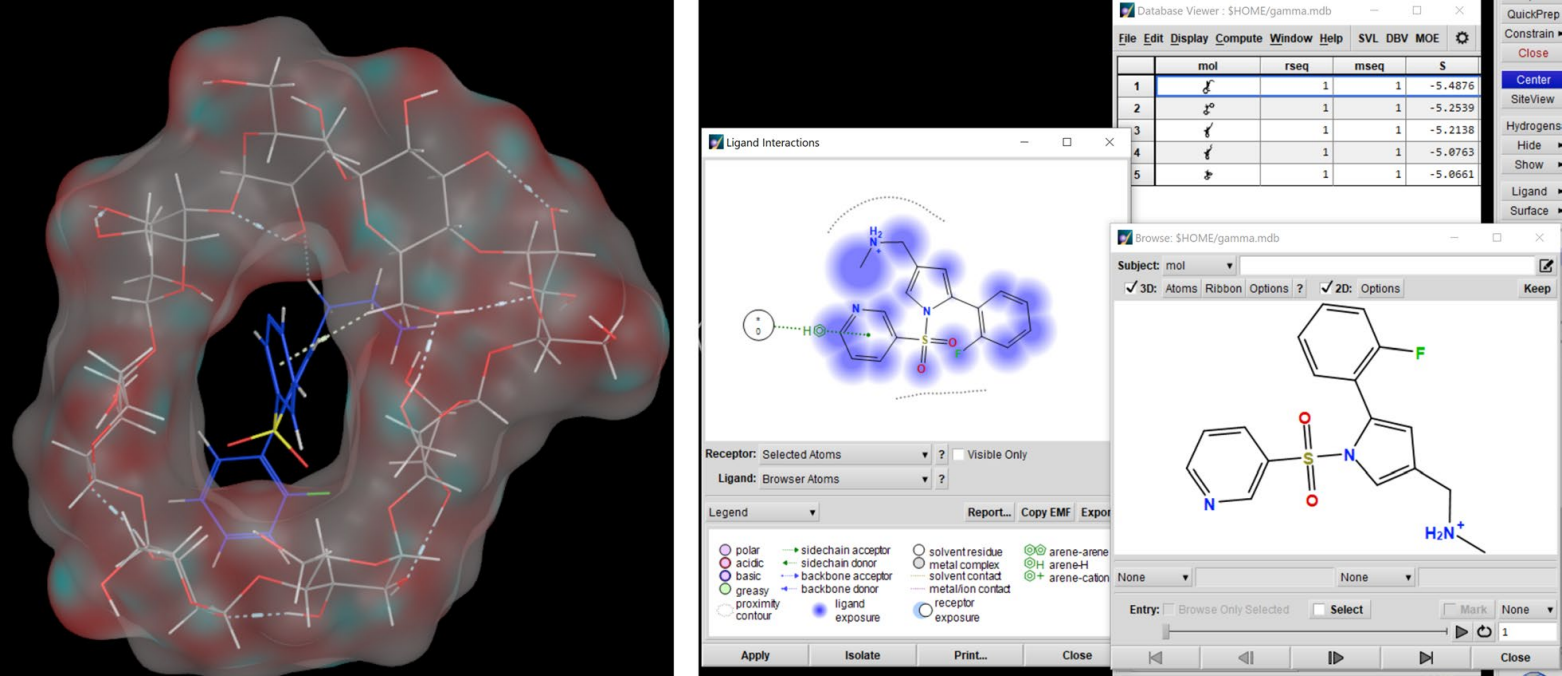
Docking result of γ-cyclodextrin

The impact of ionophore incorporation and consequently its ratios in ISM was also investigated. Molecular docking study was employed to identify the most suitable ionophore to target VON. According to the results presented in Table 2, γ-cyclodextrin exhibited the most favorable docking score (lowest S value), and that indicates the formation of a stable complex which is crucial for sensor performance. This improves ion exchange kinetics within the ISM and enhances sensitivity and reduces response time [41]. The interaction between VON and γ-cyclodextrin cavity, is illustrated in Fig. 1. The application of molecular docking proved the practical experiments in identifying the most appropriate ionophore for ISM formulation, thereby streamlining experimental efforts. This approach significantly minimizes the need for extensive trial and error experiments, reducing both chemical consumption and exposure to hazardous substances.

**Table 2:** Docking results (S value) for interaction between different ionophores and VON

Ionophore	(1st) S value
Alpha cyclodextrin	−4.07
Beta cyclodextrin	−5.04
γ-cyclodextrin	−5.49
Calix[4]arene	−3.72
Calix[6]arene	−5.03
Calix[8]arene	−4.73

#### Optimization of γ-cyclodextrin amount

The practical results revealed that the Nernstian slope and the response time were greatly enhanced upon incorporation of γ-cyclodextrin in membrane cocktail (Sensor 9) (30 s) in comparison to ionophore free membrane (Sensor 6) (40 s). The impact of the amount of γ-cyclodextrin on the composition of the ISM was investigated via preparing different membranes having different amounts of the selected ionophore over the range of 5.0 to 15.0 mg (Sensor 9–11). It was found that upon increasing the amount of γ-cyclodextrin from 5.0 to 10.0 mg the slope value of the calibration curves increased from 57.00 to 59.00 mV/decade, on the other hand when the amount of γ-cyclodextrin increased to 15.0 mg the Nernstian slope decreased to 57.50 mV/decade, so the optimal amount of γ-cyclodextrin that has the highest Nernstian slope (59.00 mV/decade) was found to be 10.0 mg (Sensor 9).

#### Effect of graphene nanocomposite transducer

**Fig. 2:**
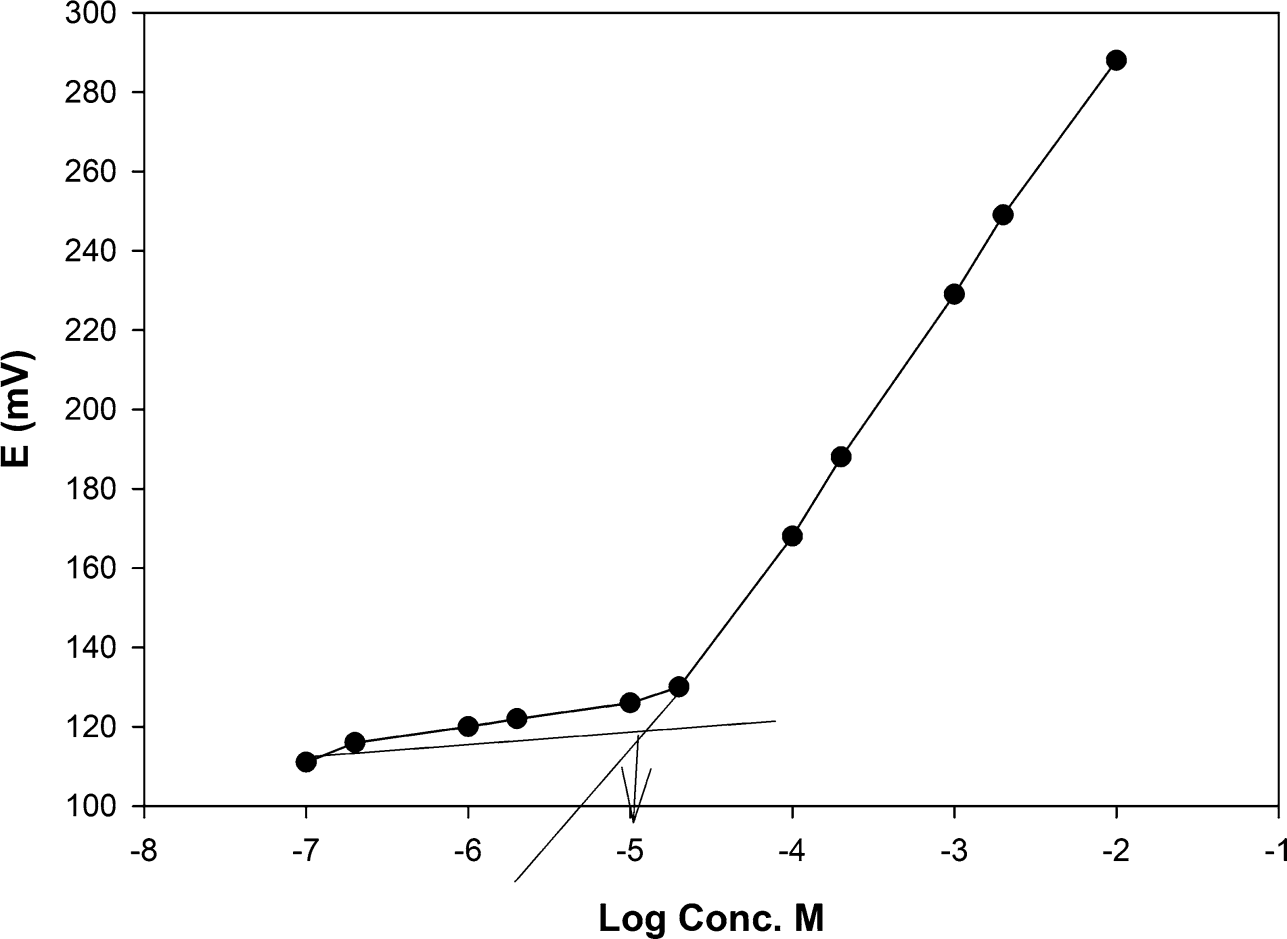
Calibration graph for determination of VON using the optimized sensor (Sensor 12)

Cu based electrodes and other solid contact ISEs commonly encounter challenges related to potential instability and signal drift. These issues predominantly arise from the inefficient coupling between the ISM and the electron conductive solid contact. Additionally, the formation of an intermediate aqueous layer at this interface can serve as an electrolyte reservoir that buffers fluctuations in sample composition. However, this water layer negatively impacts the electrode’s potential stability and degrades the sensor’s LOD by introducing additional pathways for ion flux and capacitive decoupling [31]. Consequently, the optimal sensor (Sensor 9) was modified with the graphene nano composite (Sensor 12), both two sensors (Sensor 9, 12) achieved a Nernstian slope covering a concentration range of 2$.00 \times$ 10^–5^–1.00 $\times$ 10^–2^ M and slopes of 59.00, 59.21 mV/decade respectively as illustrated in Table 3. The calibration curve for Sensor 12 is presented in Fig. 2. Sensor 12 exhibited a LOD of 1.00 × 10⁻^5^ M for VON (Table 4), which is significantly lower than that obtained with the sensor lacking the graphene nanocomposite layer (Sensor 9), which demonstrated a LOD of 1.50 × 10⁻^5^ M for VON. The reproducibility of the calibration curves for Sensors 9 and 12 was assessed by calculating the standard deviation (SD) of the slopes from three independently fabricated sensors of each type. Sensor 12 demonstrated superior reproducibility with a lower SD of 2.50, compared to a considerably higher SD of 7.60 observed for Sensor 9. These findings confirm that the incorporation of graphene as a transducer layer effectively enhanced sensor performance in terms of both detection sensitivity and reproducibility.

**Table 3:** The characteristics of potentiometric response of VON with variable constituents of the sensing layer

Sensor	Slope, mV/concentration decade	Linear range (M)	r^2^
PTA			
Sensor 1	39.00	1.00 × 10^−4^ to 1.00 × 10^−2^	0.99
Sensor 2	41.00	1.00 × 10^−4^ to 1.00 × 10^−2^	0.99
Sensor 3	50.00	1.00 × 10^−4^ to 1.00 × 10^−2^	0.9995
TETR			
Sensor 4	47.00	2.00 × 10^−5^ to 1.00 × 10^−2^	0.9991
Sensor 5	52.00	2.00 × 10^−5^ to 1.00 × 10^−2^	0.9992
Sensor 6	52.18	2.00 × 10^−5^ to 1.00 × 10^−2^	0.9961
Sensor 7	49.00	2.00 × 10^−5^ to 1.00 × 10^−2^	0.995
Sensor 8	51.00	2.00 × 10^−5^ to 1.00 × 10^−2^	0.998
Sensor 9	59.00	2.00 × 10^−5^ to 1.00 × 10^−2^	0.9991
Sensor 10	57.00	2.00 × 10^−5^ to 1.00 × 10^−2^	0.999
Sensor 11	57.5	2.00 × 10^−5^ to 1.00 × 10^−2^	0.9991
Sensor 12 (doped with graphene nanoplate)	59.21	2.00 × 10^−5^ to 1.00 × 10^−2^	0.9993

**Table 4:** Response parameters of the selected sensor (Sensor 12) for determination of VON in pure form

Parameters	Value
Slope (mV/ concentration decade)	59.21
Intercept	406.99
Square correlation coefficient (R^2^)	0.9993
Linearity range (M)	2.00 × 10^−5^ to 1.00 × 10^−2^
LOQ (M)	2.00 × 10^−5^
LOD (M)	1.00 × 10^−5^
%Mean recovery (±SD)	100.00 ± 0.72
Relative standard deviation (%RSD)	0.72

### Characterization of the microfabricated Cu electrode and graphene transducer

The stability of all solid contact ISEs is critically dependent on the quality of the solid-contact transducer layer, particularly its ability to prevent the formation of an intermediate aqueous layer and ensure efficient ion-to-electron transfer. As Cu is prone to oxidation, which can destabilize the potential, we performed comprehensive elemental and morphological characterization to confirm the efficacy of the cleaning and modification steps.

#### Surface purity and oxide removal

**Fig. 3:**
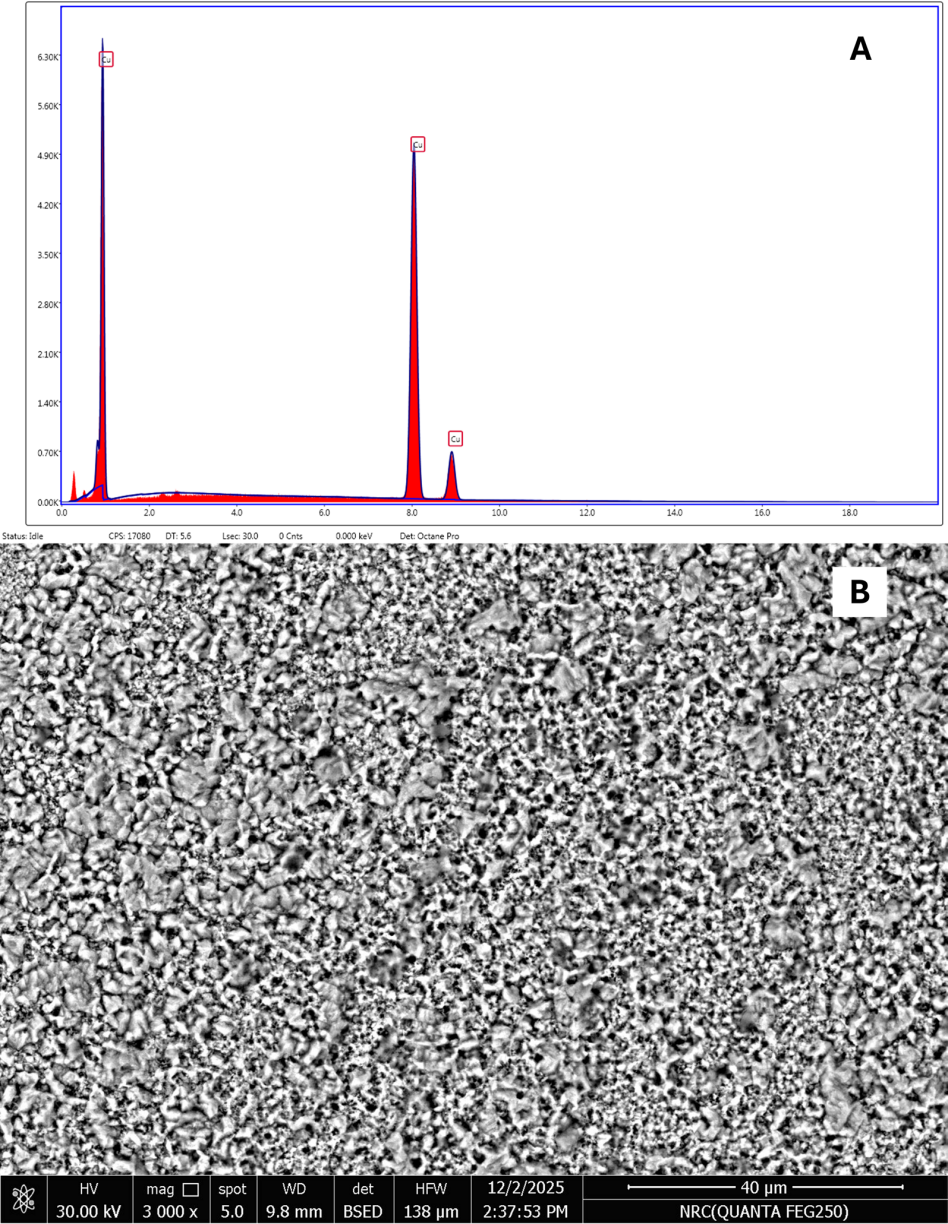
**A** EDS, **B** SEM image of bare Cu electrode

Energy-dispersive X-ray spectroscopy (EDS) was employed to verify the purity of the microfabricated Cu electrode immediately after the final cleaning protocol. This analysis was necessary to confirm the complete removal of any native or process-induced Copper oxide residues. The EDS spectrum of the bare Cu surface displayed peaks exclusively for Cu with the elemental composition of 99.85% Cu by weight (Fig. 3). Crucially, there was a complete absence of any detectable Oxygen peak, providing quantitative evidence that the sequential washes with acetone and glacial acetic acid were effective in preparing a pristine, non-oxidized Cu surface.

#### Morphological and elemental integrity of the graphene transducer

**Fig. 4:**
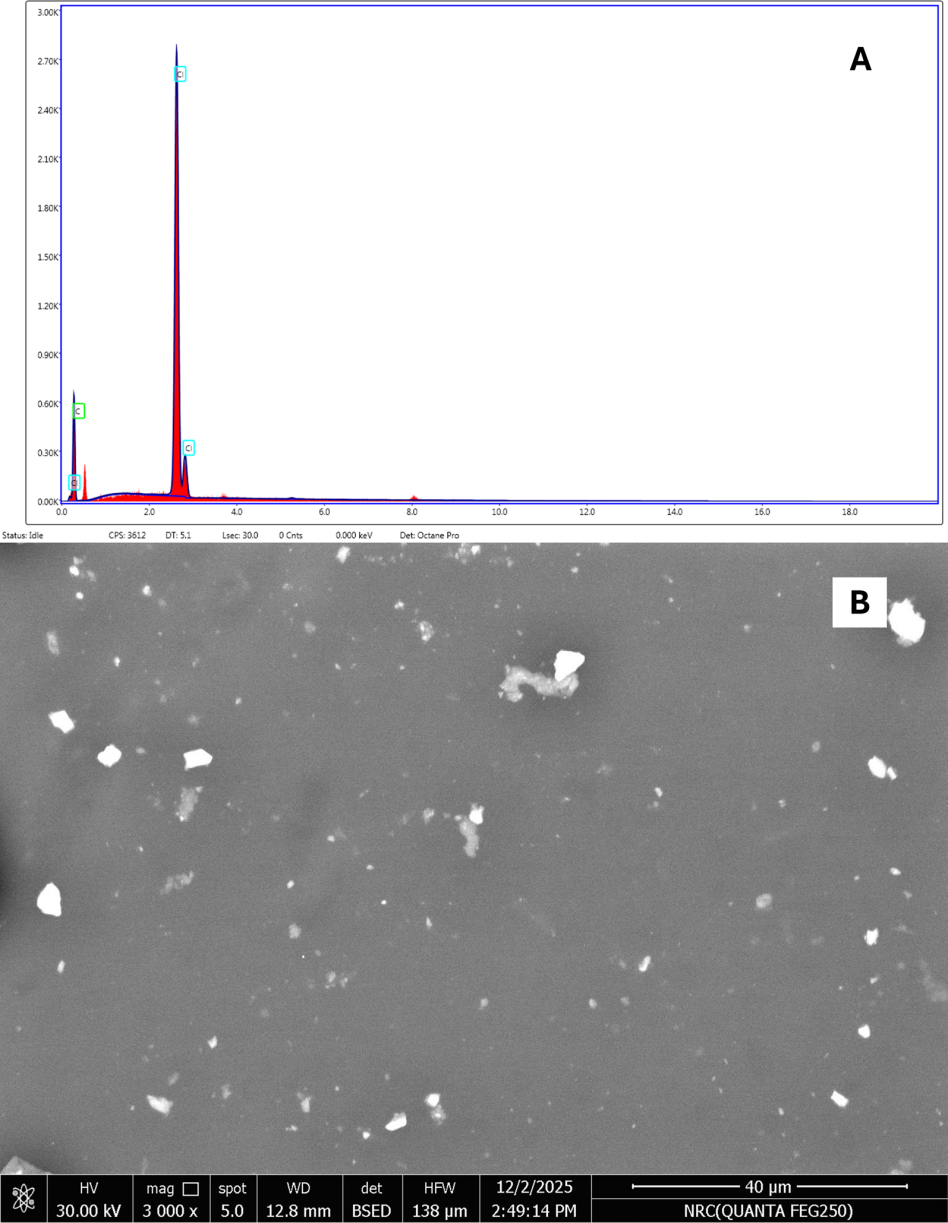
**A** EDS, **B** SEM image of graphene-coated electrode

The optimized Cu electrode was subsequently coated with the Graphene/PVC nanocomposite layer. This layer functions as both the ion to electron transducer and the hydrophobic barrier that isolates the Cu from the ISM. Scanning Electron Microscopy (SEM) images confirmed the uniform, dense, and non-porous surface morphology of the drop casted graphene/PVC layer (Fig. 4A). The absence of observable pinholes or defects across the entire Cu track visually confirms that the layer is continuous, thereby ensuring maximum protection against water ingress and preventing aqueous layer formation. Elemental analysis of the nanocomposite surface (Fig. 4B) showed dominant peaks for Carbon and expected signals from Chlorine (originating from the PVC). The signal from the underlying Cu was significantly attenuated or absent in the focused area, further indicating a uniform coverage with sufficient thickness. The successful characterization confirms that the Cu substrate is clean and fully covered by a robust, non-porous, and conductive graphene layer, directly validating the structural design necessary to achieve the low potential drift and high reproducibility observed in the subsequent potentiometric measurements.

### Effect of pH

**Fig. 5:**
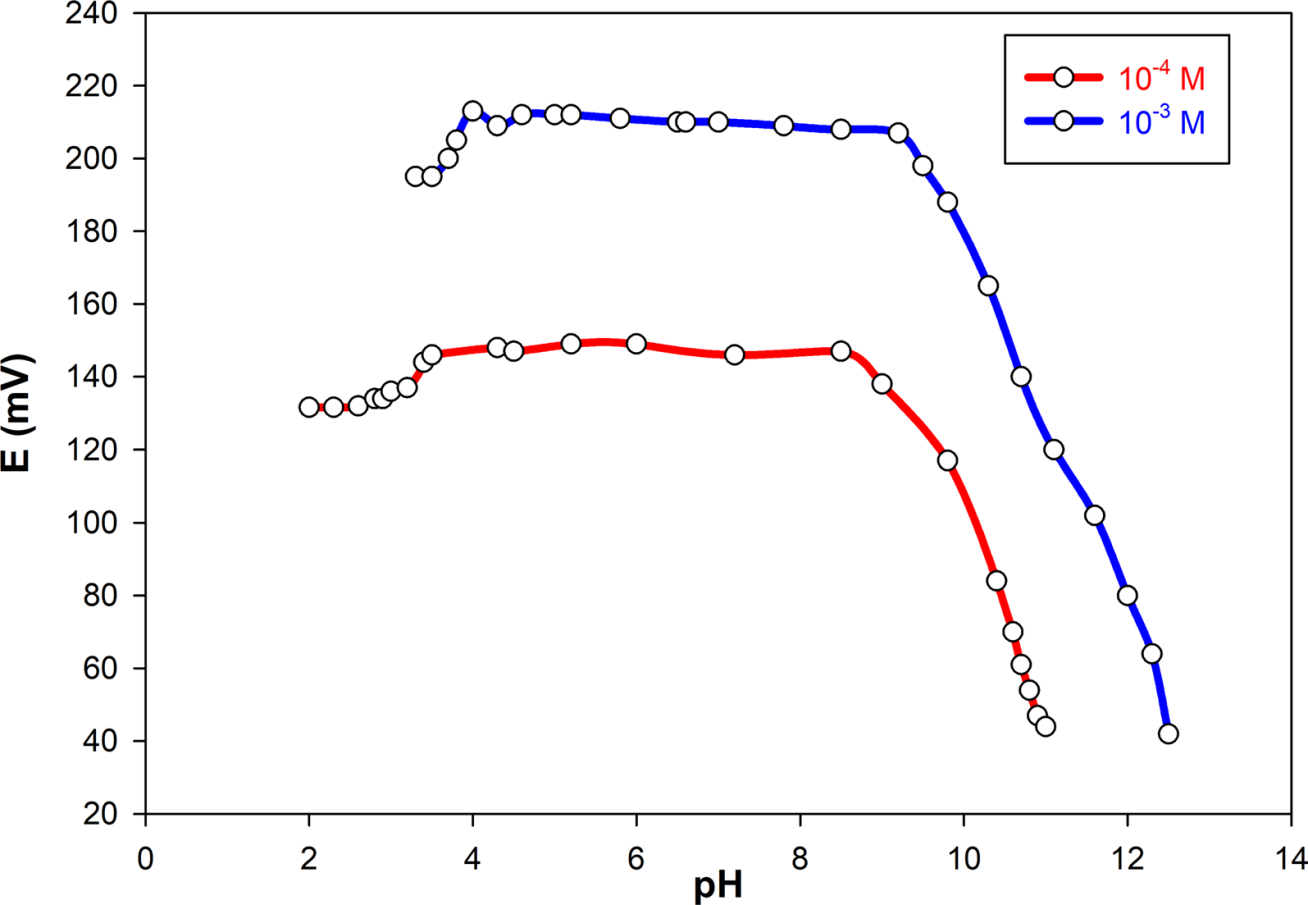
The effect of pH on the response of sensor 12 using 1.00 × 10^–4^ and 1.00 × 10^–3^ M VON solutions

Establishing the electrode potential within a pH range that ensures the measured response is solely dependent on the concentration of VON and unaffected by pH fluctuations is essential. To evaluate this, the pH dependent behavior of the selected sensor (Sensor 12) was assessed at two concentrations of VON: 1.00 × 10⁻^4^ and 1.00 × 10⁻^3^ M. The pH of the test solutions was systematically adjusted using either HCl or NaOH. Results indicated that within the pH range of 4.00 to 7.00, the electrode exhibited a stable and consistent potential, demonstrating independence from pH variation. Below pH 4.00, minor deviations in potential were detected, likely due to interference from proton (H⁺) activity. Conversely, at pH levels exceeding 7.00, pronounced shifts in emf were observed, as depicted in Fig. 5. Based on these findings, all subsequent measurements were conducted in phosphate buffer solutions maintained at pH 5.00 ± 0.05 to ensure optimal sensor performance and reproducibility.

### Hysteresis and reproducibility

**Fig. 6:**
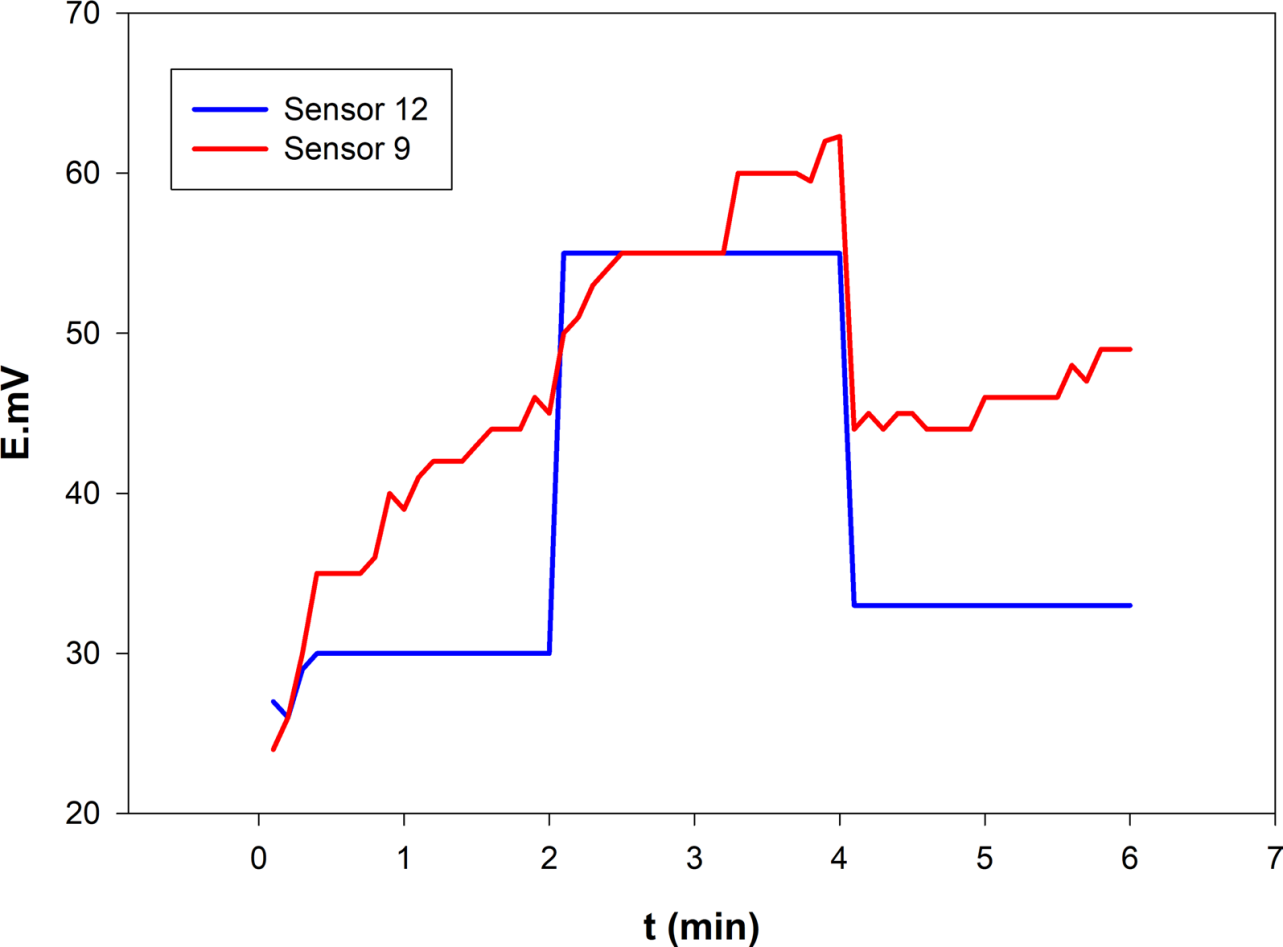
Potential reproducibility of the selected sensor (Sensor 12) compared to (Sensor 9) when exposed to 1.00 × 10^−4^ M VON and 1.00 × 10^−3^ M VON

The formation of an aqueous layer is a pivotal factor influencing potential drift in microfabricated ISEs, with such hysteresis effects closely linked to the development of this water layer. In this study, hysteresis assessments were performed on the optimized microfabricated sensor (Sensor 12) and its graphene-free counterpart (Sensor 9). According to IUPAC, hysteresis denotes the variance in electrode potential measured for identical analyte concentrations after exposure to solutions of varying concentrations. To evaluate hysteresis, both Sensors 12 and 9 were initially immersed in a 1.00 × 10⁻^4^ M solution for 2 h, subsequently transferred to a 1.00 × 10⁻^3^ M solution for another 2 h, and finally returned to the initial solution for an additional hour. As depicted in Fig. 6, Sensor 12, which incorporates hydrophobic graphene, exhibited minimal potential drift, indicating the absence of a water layer. In contrast, Sensor 9, lacking graphene, demonstrated significant potential drift, suggestive of water layer formation at the solid-contact interface. Graphene's remarkable conductivity, electrocatalytic activity, and hydrophobic nature make it an ideal ion-to-electron transducer. The graphene free ISM γ-cyclodextrin sensor (Sensor 9) exhibited a potential drift of 10.50 mV/h in a 1.00 × 10⁻^4^ M VON solution. This drift was substantially reduced to 3.00 mV/h with the incorporation of graphene (Sensor 12), aligning with previous studies that highlight graphene's effectiveness in preventing water layer formation beneath the ISM.

### Selectivity of the optimized sensor

The selectivity of the optimized sensor (Sensor 12) for VON was systematically evaluated using the separate solution method [47], in the presence of various potentially interfering drugs, co administered drugs, most interfering excipients, and cations. Selectivity coefficients were determined for each interfering species and are presented in Table 5. The findings demonstrated that the incorporation of γ-cyclodextrin as an ionophore in sensor 12 significantly enhanced membrane selectivity toward VON when compared to the ionophore free sensor (Sensor 6). This enhancement is in line with the molecular docking results.

**Table 5:** Selectivity coefficients of the sensor 6 and 12 via the separate solution method

Interferents	log K^Pot^_primary ion, interferent_ Sensor 6	log K^Pot^_primary ion, interferent_ Sensor 12
Metoclopramide	−1.80	−2.80
Tolperisone HCl	−2.98	−3.40
Diclofenac sodium	−2.09	−3.02
Amoxicillin	−1.90	−2.40
Stearate	−2.90	−3.63
Mg^++^	−4.07	−4.98
Na^+^	−4.64	−5.13
K^+^	−5.23	−6.11
Ca^++^	−3.42	−4.31

### Potentiometric determination of VON in tablet dosage form

The optimized sensor (Sensor 12) was employed for the quantitative analysis of VON in its commercial tablet formulation (Vonaspire® tablet). The developed sensor demonstrated high analytical performance with good accuracy and precision, as respectively reflected by high percentage recoveries and low SD values presented in Table 6.

**Table 6:** Estimation of VON in its tablet dosage form by the developed potentiometric method and reported method, compared using Student’s *t*-test and *F*-test

Dosage form	Developed	Reported [7]
Mean % recovery ± SD	99.54 ± 0.52	98.76 ± 0.78
N	3	3
Student's *t*-test (2.78)	0.23	
F test (19.00)	2.27	

### Evaluation of the developed method through statistical comparison with the reported method

To validate the reliability of the developed method, a statistical comparison was made between the results obtained using Sensor 12 and those from the reported method [7]. Application of the Student’s *t*-test and *F*-test revealed no statistically significant differences between the two methods at a 95% confidence level (*p* = 0.05) [48], confirming accuracy and precision of the proposed approach, as shown in Table 6.

### Greenness assessment of the analytical method

**Fig. 7:**
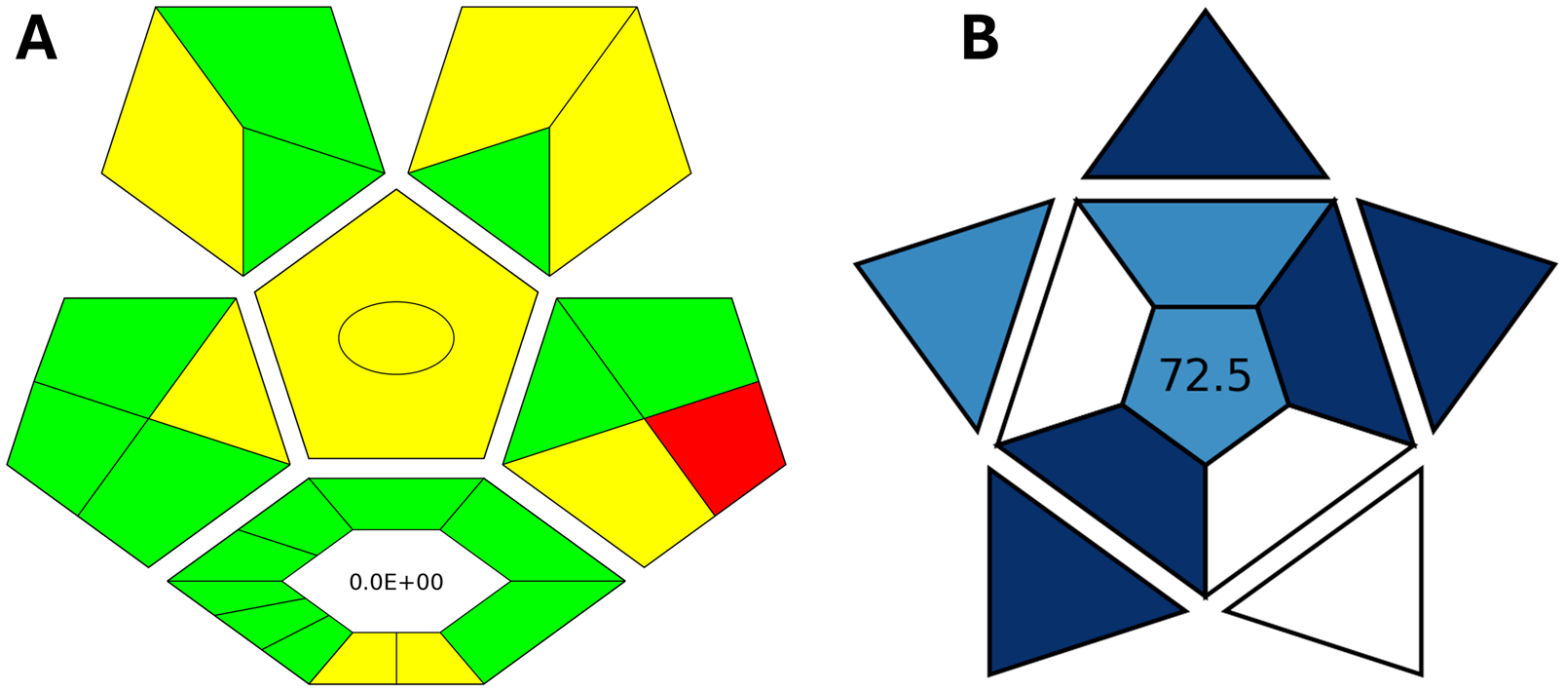
The greenness assessment of the developed method using Complex-GAPI (**A**) and BAGI (**B**) tools

In alignment with the principles of green and sustainable chemistry, which aim to reduce hazardous waste generation globally, it was essential to evaluate the environmental impact of the proposed analytical method. The greenness of the method was assessed using Complex-GAPI [49], which provides a comprehensive evaluation of the entire analytical workflow, beginning with the pre-analytical phase. This assessment is visually represented by a hexagonal diagram, where green indicates eco-friendly steps, yellow denotes moderate environmental impact, and red highlights environmentally hazardous steps. As shown in Fig. 7A, the only red segment in the Complex-GAPI profile corresponds to the waste volume produced during the analysis. However, this waste can be effectively treated and neutralized, thereby minimizing its environmental risk. In addition to green assessment. Blue applicability grade index (BAGI) a recently developed metric for evaluating the practical applicability of analytical methods was also employed [50]. This index uses shades of blue to indicate method feasibility: dark blue for high applicability, blue for moderate, and light blue for low. A BAGI score above 60.0 is indicative of a method with acceptable practical utility. The evaluated method achieved a score of 72.5 (Fig. 7B), confirming its strong practicality and operational feasibility.

## Conclusion

This research demonstrates the development of a microfabricated ISE tailored for the detection of VON, aligning with the principles of green analytical chemistry and advancing portable analytical technologies. The study uniquely identifies γ-cyclodextrin as the optimal ionophore through molecular docking studies. Additionally, TETR emerged as the best alternative IP, while o-NPOE was identified as the most effective plasticizer in enhancing sensor performance. The incorporation of a graphene nanocomposite as an ion to electron transducer layer significantly improved the stability of the sensor, minimized potential drift, and ensured rapid response times (~30 s). This enhancement is attributed to the hydrophobic nature of the nanocomposite, which prevents water layer formation at the interface between the Cu electrode and the polymeric membrane. The optimized sensor exhibited remarkable analytical performance, characterized by a linear dynamic range of 2.00 × 10⁻^5^ to 1.00 × 10⁻^2^ M and a LOD of 1.00 × 10⁻^5^ M, adhering to IUPAC guidelines. Its applicability was validated through the selective determination of VON pharmaceutical formulations with statistical analysis confirming no significant difference compared to the reported method. Furthermore, the environmental impact of the developed method was rigorously assessed using Complex-GAPI and BAGI, underscoring their eco-friendly and sustainable attributes. By leveraging microfabrication techniques and graphene-based transducers, this research not only addresses the growing need for efficient drug analysis but also supports the global shift toward green analytical practices and point-of-care applications. This study paves the way for future developments in this field, offering a robust platform for the detection of pharmaceutical compounds with high sensitivity, selectivity, and minimal environmental footprint.

## Supplementary Information

Supplementary Material 1.

## Data Availability

All the data associated with this research has been presented in this paper.

## Abbreviations

ISE: Ion selective electrode
VON: Vonoprazan fumarate
γ-cyclodextrin: Gamma-cyclodextrin
Cu: Copper
LOD: Limit of detection
PVC: Polyvinyl chloride
ISM: Ion selective membrane
DBP: Dibutyl phthalate
o-NPOE: Ortho-nitrophenyl octyl ether
DOP: Dioctyl phthalate
TETR: Potassium tetrakis(–chlorophenyl) borate
THF: Tetrahydrofuran
NaOH: Sodium hydroxide
PTA: Phosphotungstic acid
TPB: Sodium tetraphenylborate
PCBs: Photoresist-coated printed circuit boards
IP: Ion pair
