# Supplementary material for: Novel and sustainable microfabricated Cu ion selective sensor doped with ionophore and supported with docking study for determination of vonoprazan fumarate in tablet dosage form
Source: BMC Chem. 2026 Jan 30;20(1):36. doi: 10.1186/s13065-025-01714-9 (PMC12930549; doi:10.1186/s13065-025-01714-9)
Supplement: Supplementary file 1 — Supplementary Material 1. [file 13065_2025_1714_MOESM1_ESM.docx]

# Novel & Sustainable Microfabricated Cu Ion Selective Sensor Doped with Ionophore and Supported with Docking Study for Determination of Vonoprazan Fumarate in Tablet Dosage Form.

**Helal Zaher^1*^, Emad Ramzy^2^, Amr M. Mahmoud^3^, Mona S. Elshahed^2*^, Radwan EL-Haggar^1^.**

^1^ Pharmaceutical Chemistry Department, Faculty of Pharmacy, Helwan University, Ein Helwan 11795, Cairo, Egypt.

^2^ Analytical Chemistry Department, Faculty of Pharmacy, Helwan University, Ein Helwan 11795, Cairo, Egypt.

^3^ Analytical Chemistry Department, Faculty of Pharmacy, Cairo University, Kasr El-Aini Street, Cairo 11562, Egypt.
^*^ Corresponding author at Faculty of Pharmacy, Helwan University, Ein Helwan, 11795, Cairo, Egypt.

E-mail: [Helal.zaher@pharm.helwan.edu.eg](mailto:Helal.zaher@pharm.helwan.edu.eg), [mona.elshahed@pharm.helwan.edu.eg](mailto:mona.elshahed@pharm.helwan.edu.eg)

**Supporting Information**


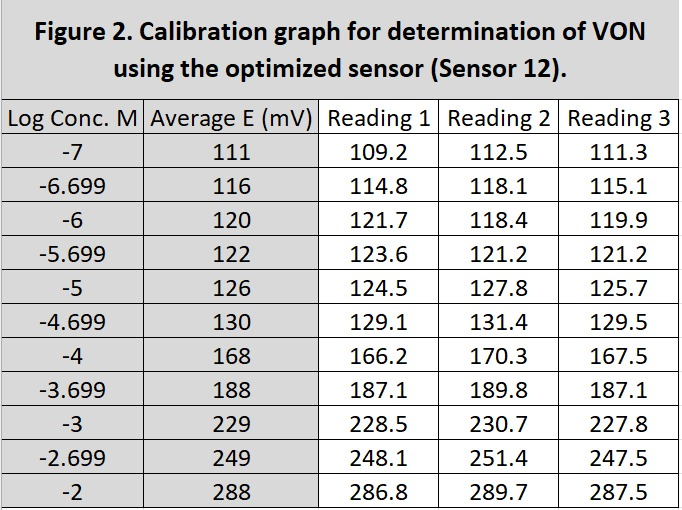


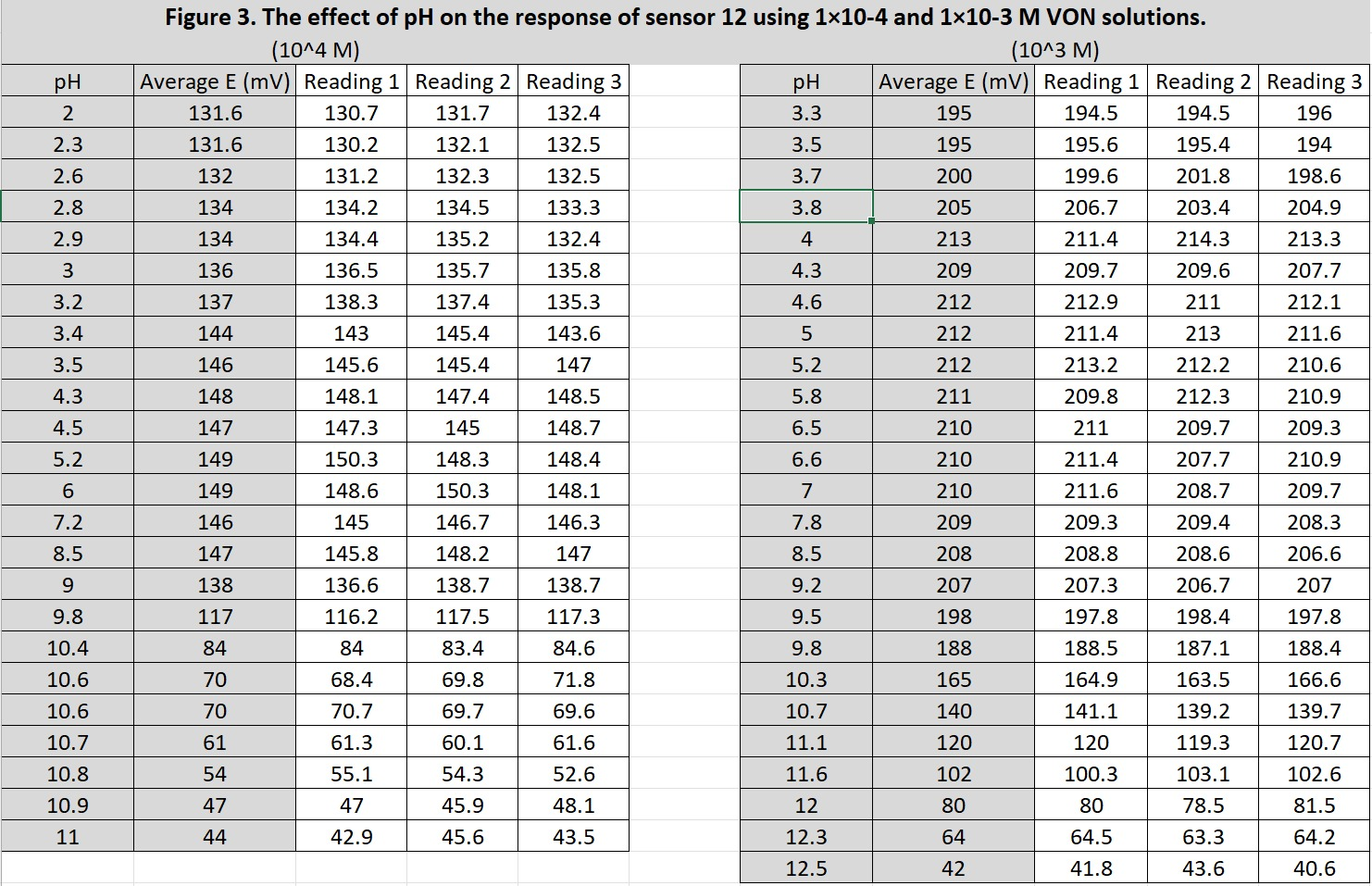


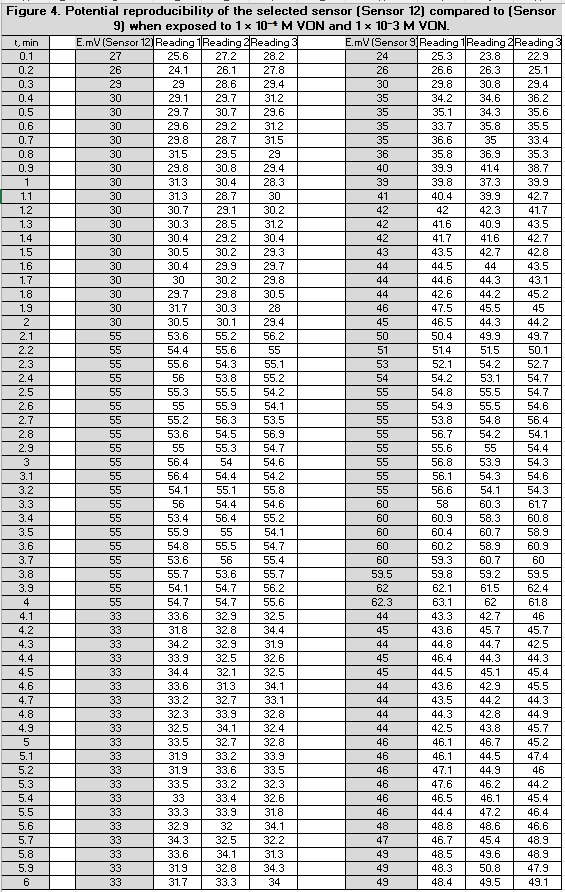


**Docking Results**

**Alpha cyclodextrine**


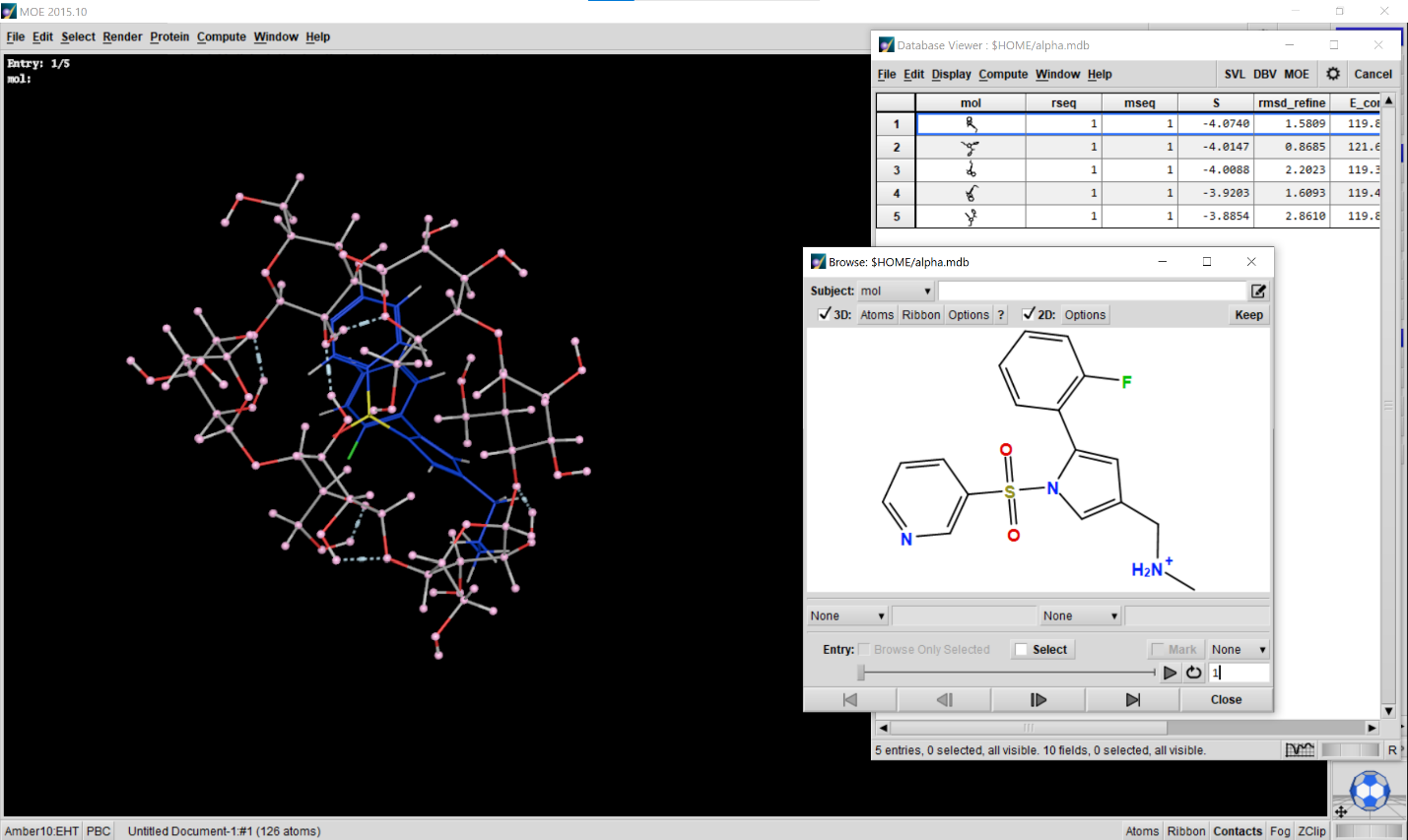

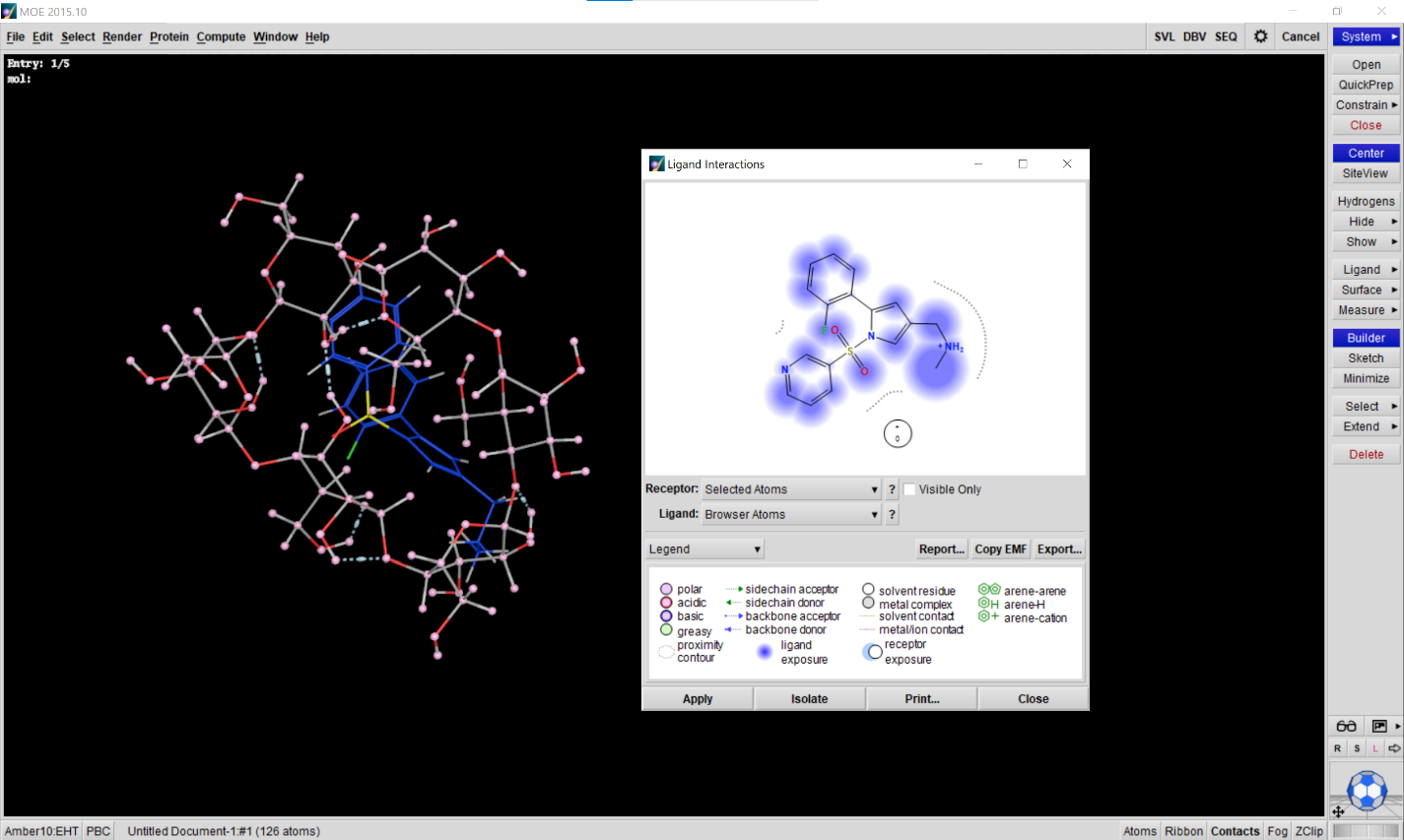


**Beta cyclodextrine**

**
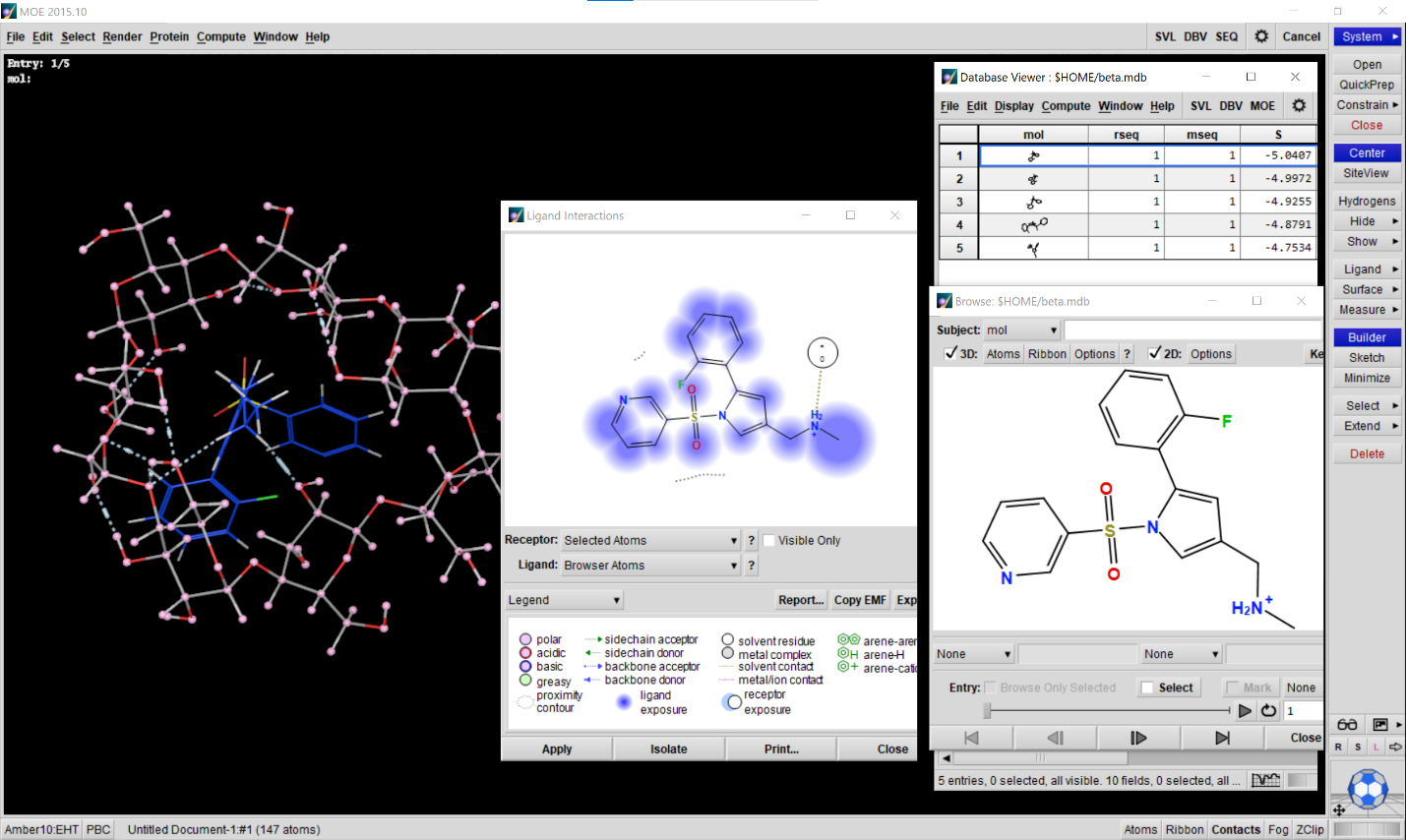

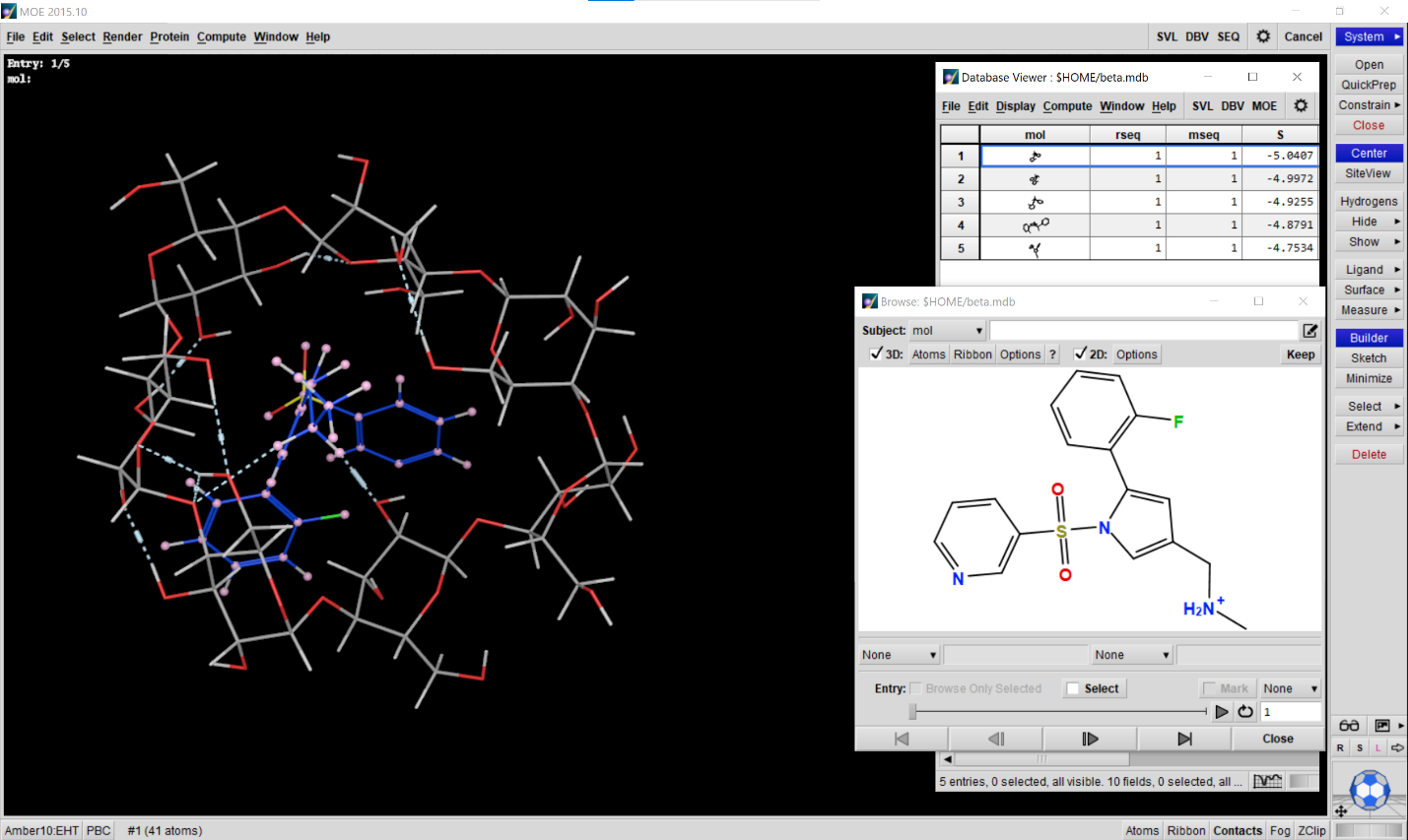
**

**Gamma cyclodextrine**

**
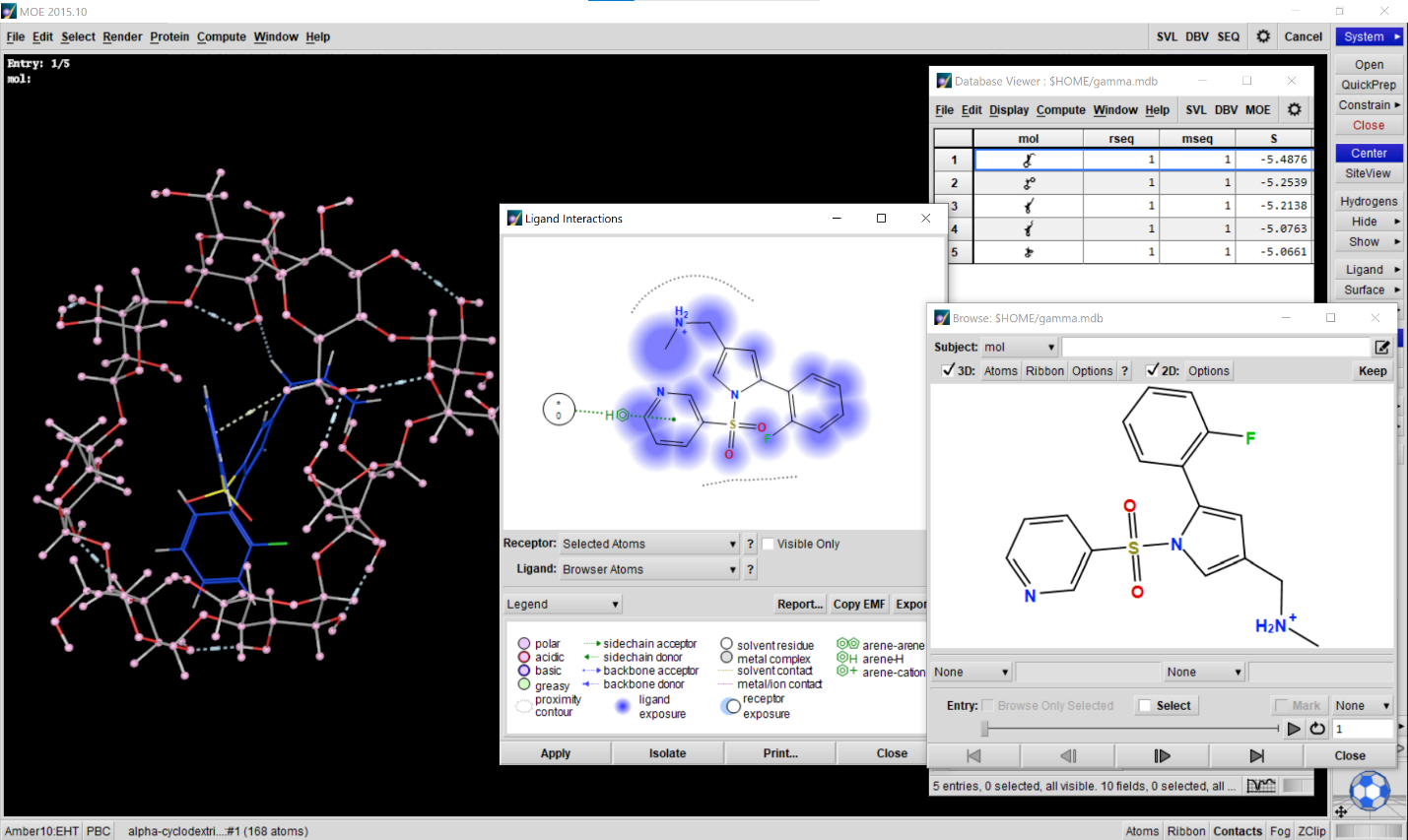

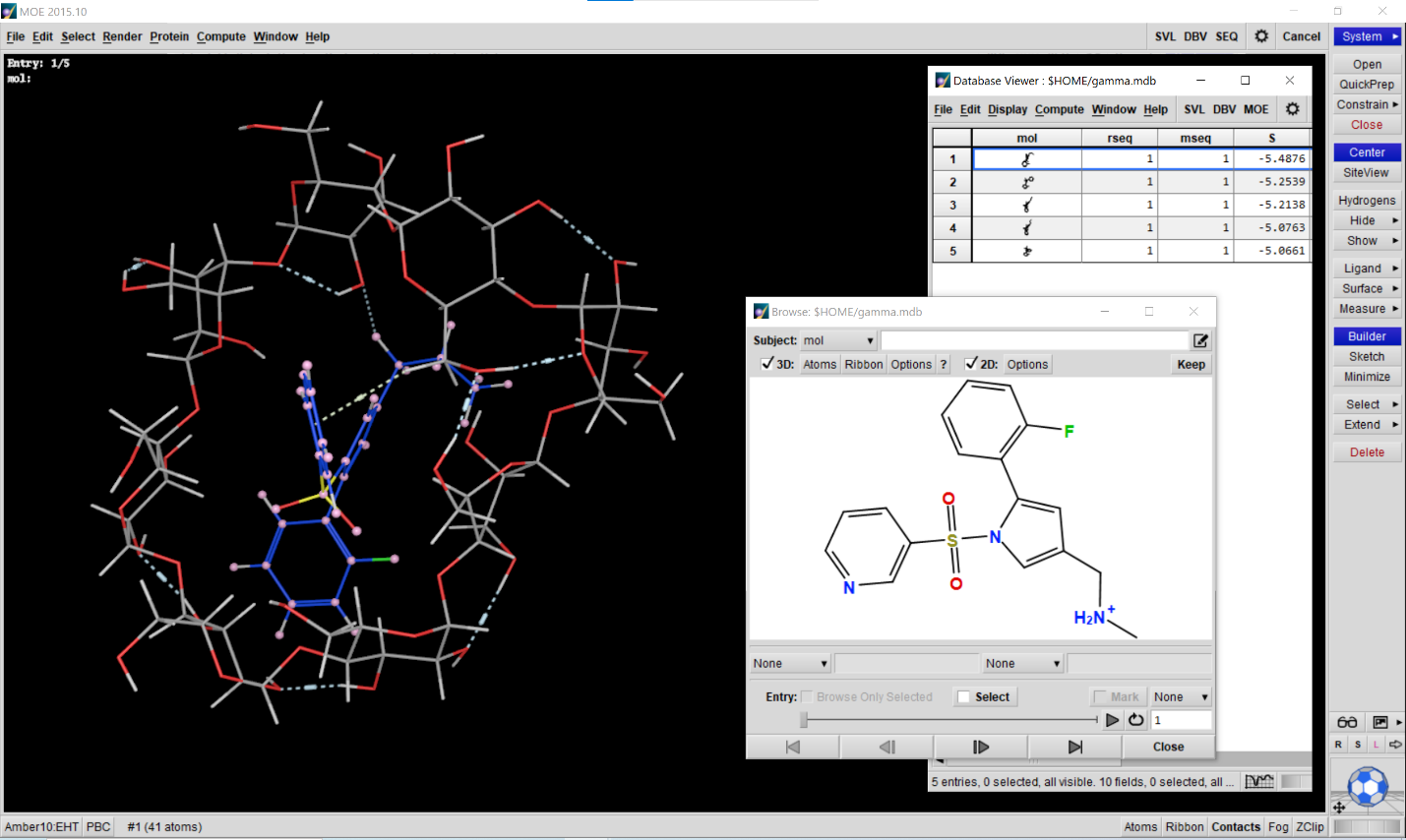
**

**Calix[4]arene**

**
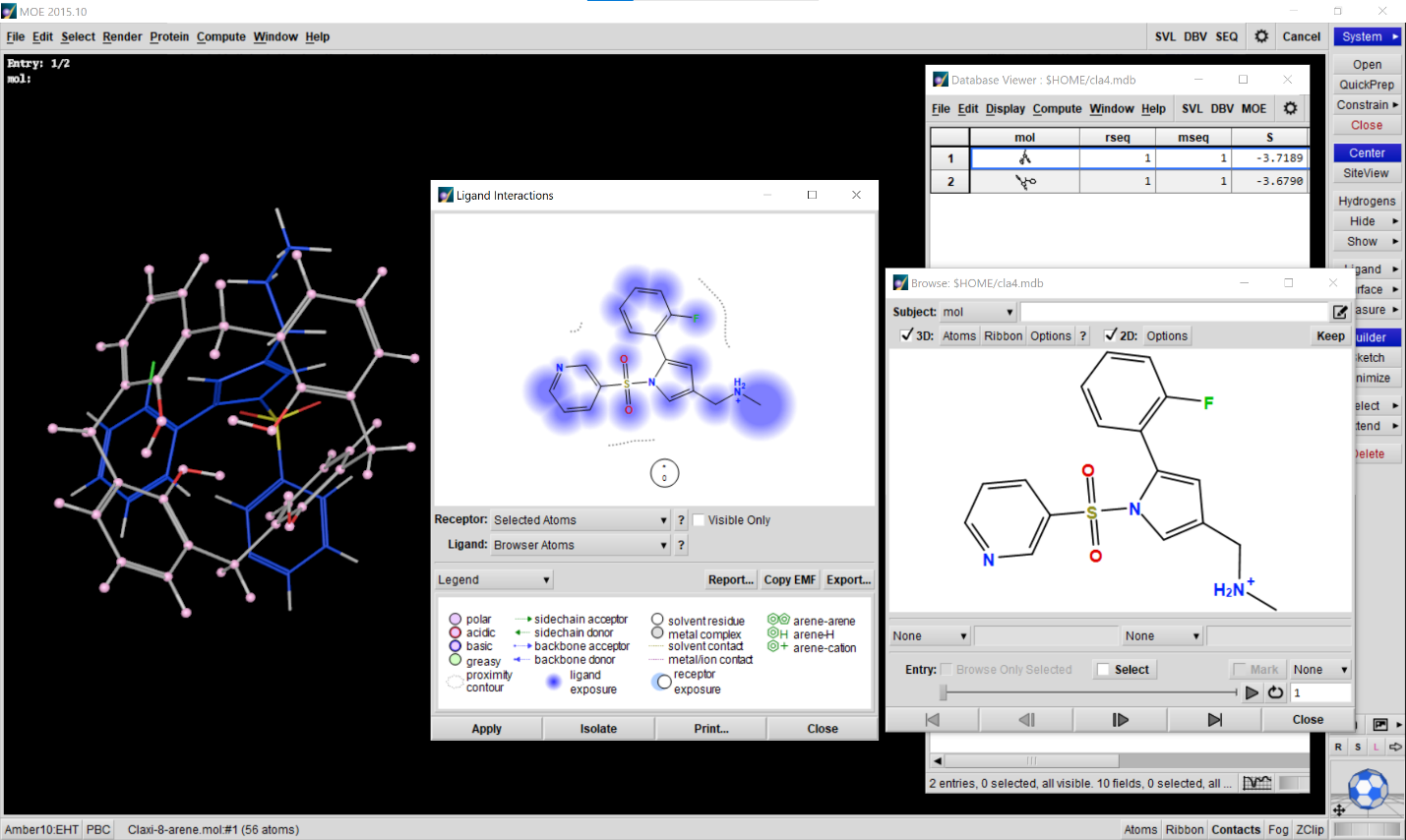

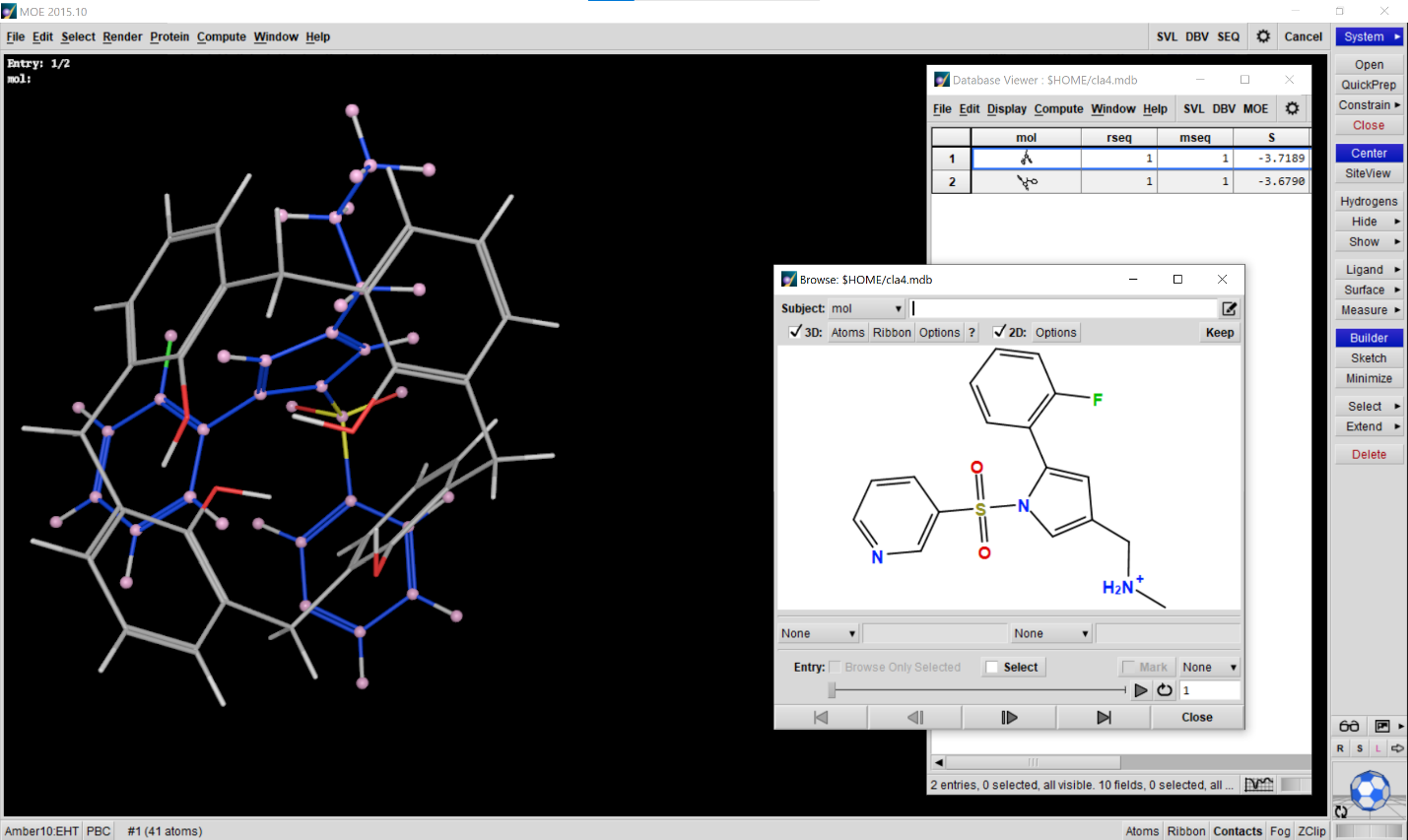
**

**Calix[6]arene**

**
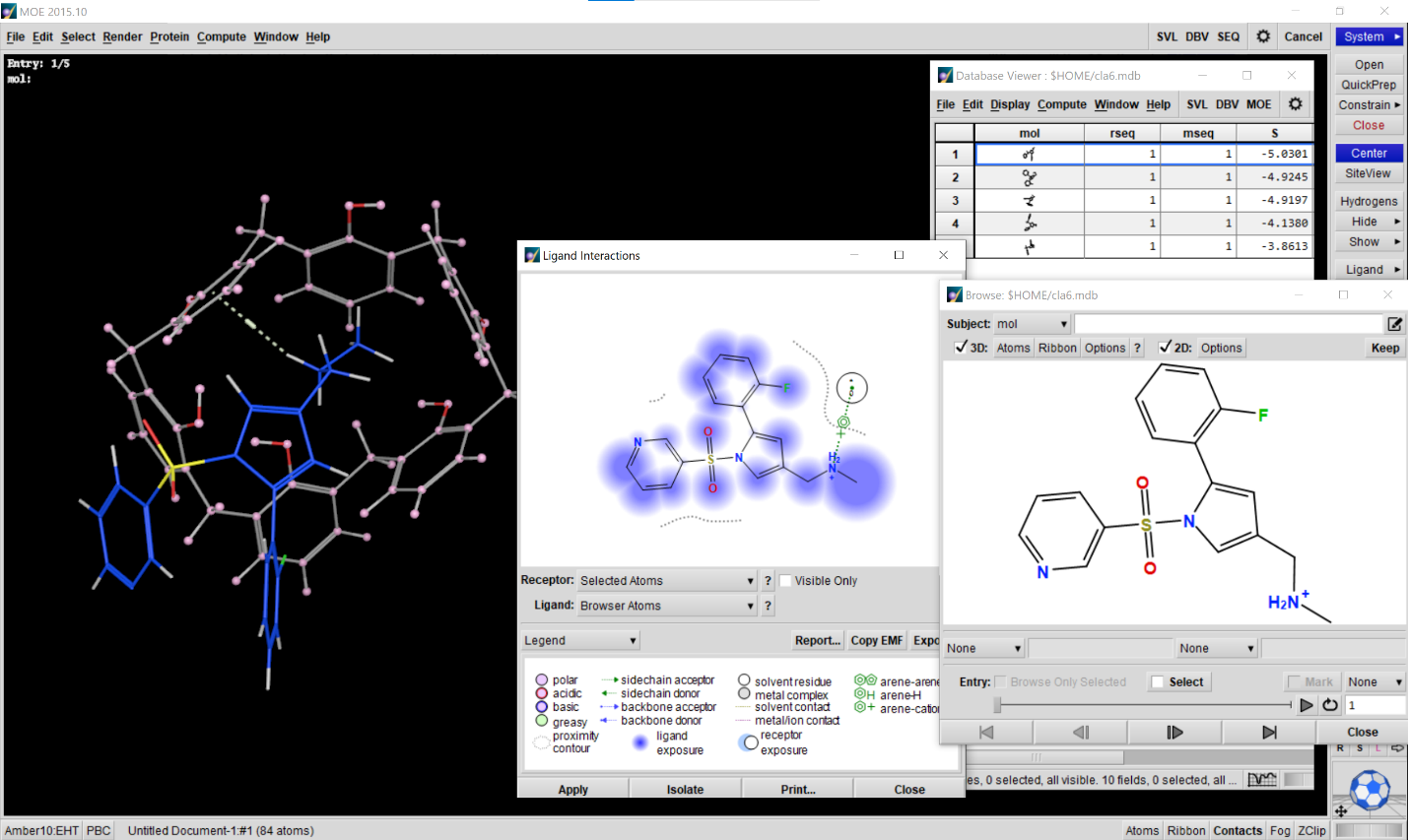

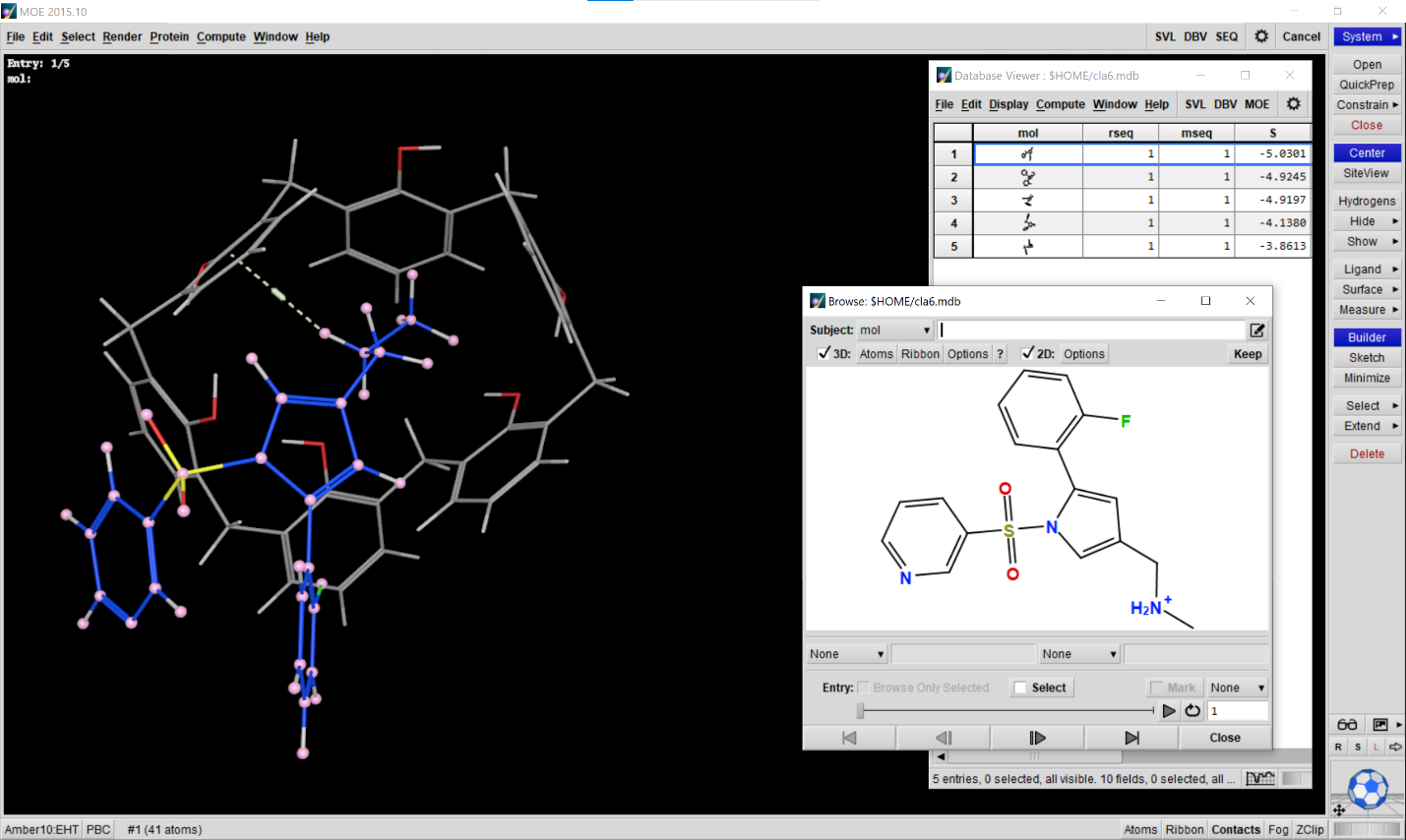
**

**Calix[8]arene**

**
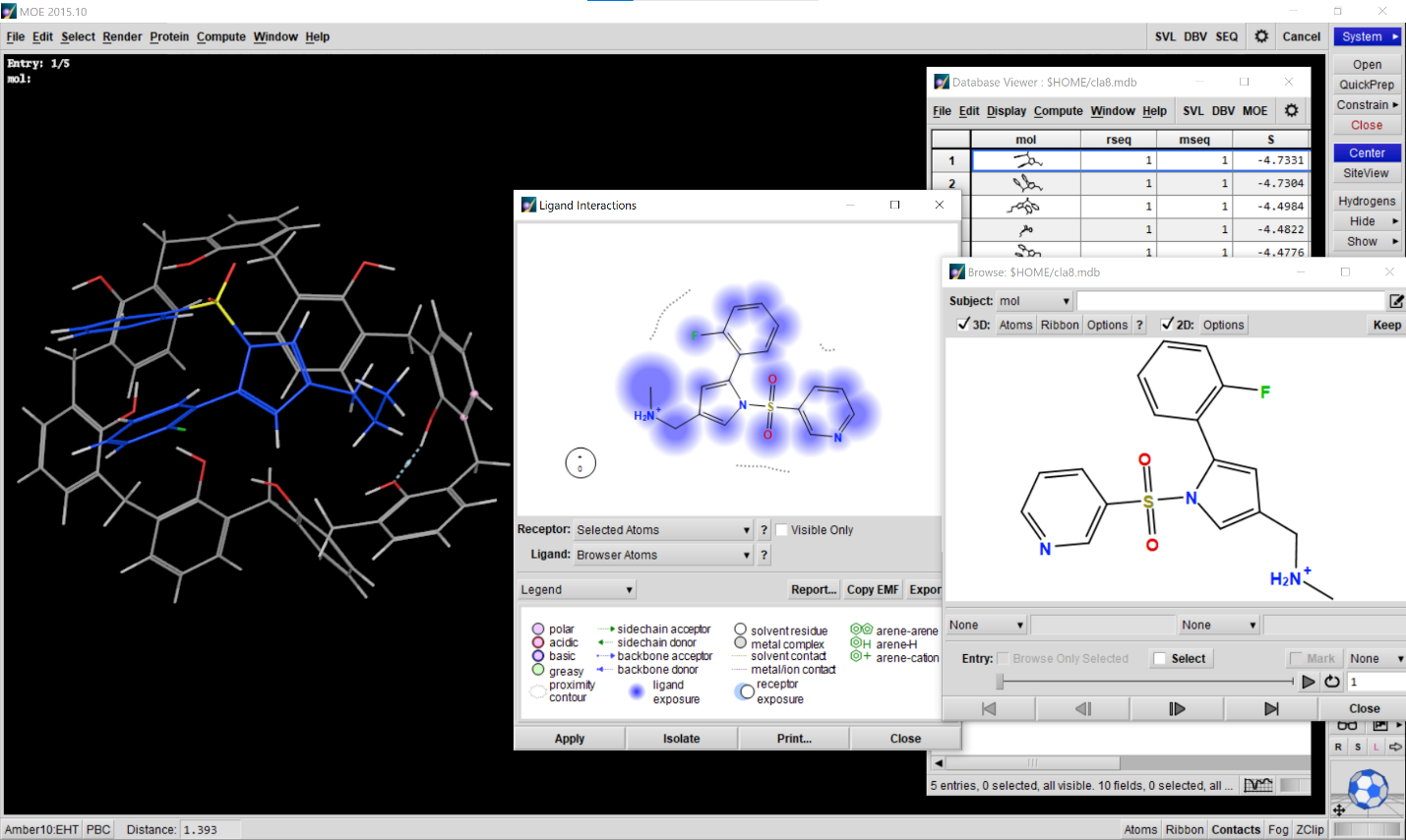

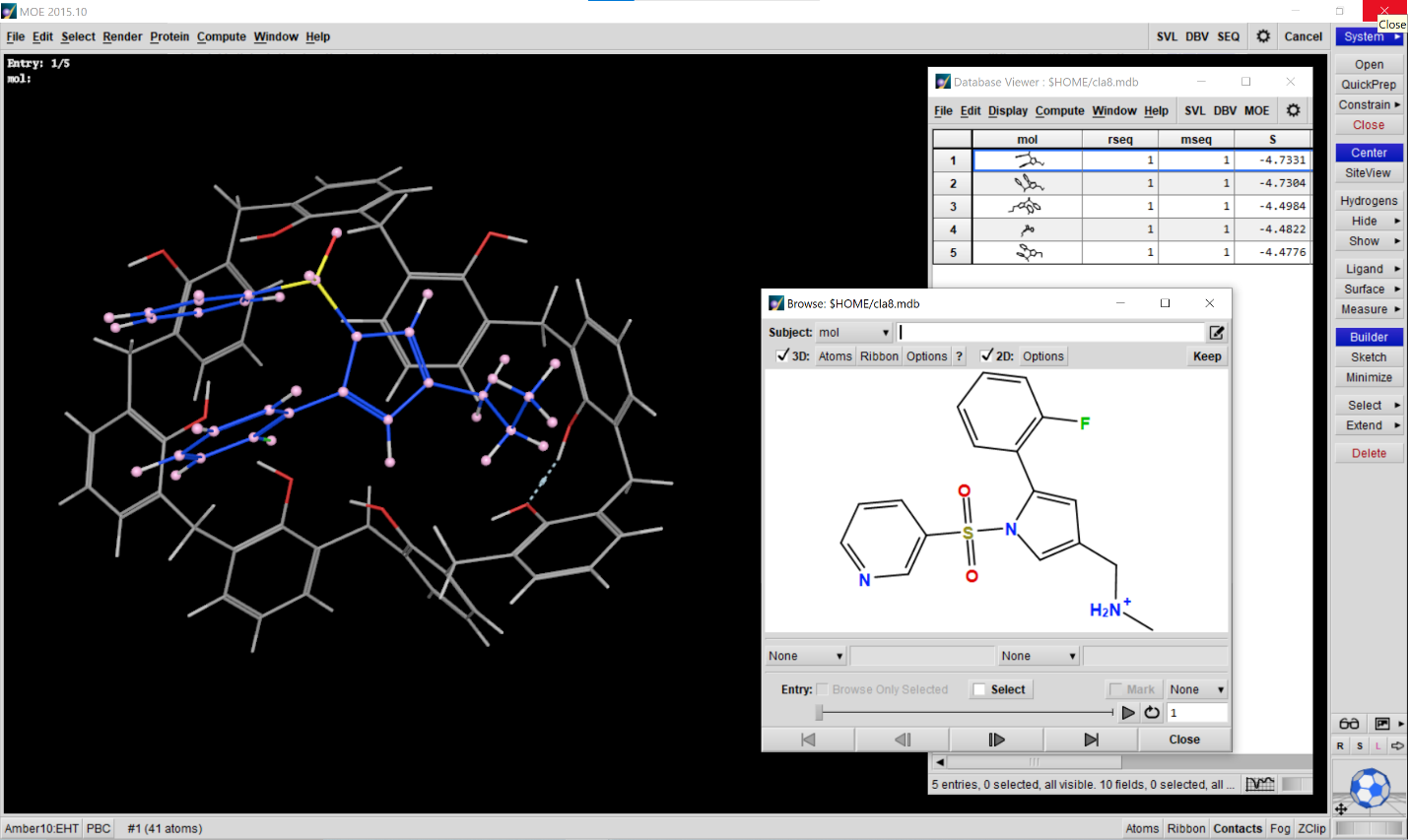
**
